# Lung cancer cell-derived exosomes: progress on pivotal role and its application in diagnostic and therapeutic potential

**DOI:** 10.3389/fonc.2024.1459178

**Published:** 2024-10-11

**Authors:** Aimi Syamima Abdul Manap, Faith Malambo Ngwenya, Meilarshny Kalai Selvan, Syarafina Arni, Fathimath Hishma Hassan, Ammar Danish Mohd Rudy, Nurul Nadiah Abdul Razak

**Affiliations:** ^1^ Department of Biomedical Science, College of Veterinary Medicine, King Faisal University, Al-Ahsa, Saudi Arabia; ^2^ Faculty of Medicine, Bioscience and Nursing, MAHSA University, Jenjarom, Malaysia; ^3^ Centre for Foundation in Science, University of Malaya, Kuala Lumpur, Malaysia

**Keywords:** lung cancer, exosome, diagnostic, therapeutic potential, lung cancer cell-derived exosomes

## Abstract

Lung cancer is frequently detected in an advanced stage and has an unfavourable prognosis. Conventional therapies are ineffective for the treatment of metastatic lung cancer. While certain molecular targets have been identified as having a positive response, the absence of appropriate drug carriers prevents their effective utilization. Lung cancer cell-derived exosomes (LCCDEs) have gained attention for their involvement in the development of cancer, as well as their potential for use in diagnosing, treating, and predicting the outcome of lung cancer. This is due to their biological roles and their inherent ability to transport biomolecules from the donor cells. Lung cancer-associated cell-derived extracellular vesicles (LCCDEVs) have the ability to enhance cell proliferation and metastasis, influence angiogenesis, regulate immune responses against tumours during the development of lung cancer, control drug resistance in lung cancer treatment, and are increasingly recognised as a crucial element in liquid biopsy evaluations for the detection of lung cancer. Therapeutic exosomes, which possess inherent intercellular communication capabilities, are increasingly recognised as effective vehicles for targeted drug delivery in precision medicine for tumours. This is due to their exceptional biocompatibility, minimal immunogenicity, low toxicity, prolonged circulation in the bloodstream, biodegradability, and ability to traverse different biological barriers. Currently, multiple studies are being conducted to create new means of diagnosing and predicting outcomes using LCCDEs, as well as to develop techniques for utilizing exosomes as effective carriers for medication delivery. This paper provides an overview of the current state of lung cancer and the wide range of applications of LCCDEs. The encouraging findings and technologies suggest that the utilization of LCCDEs holds promise for the clinical treatment of lung cancer patients.

## 1 Introduction

**Figure 1:**
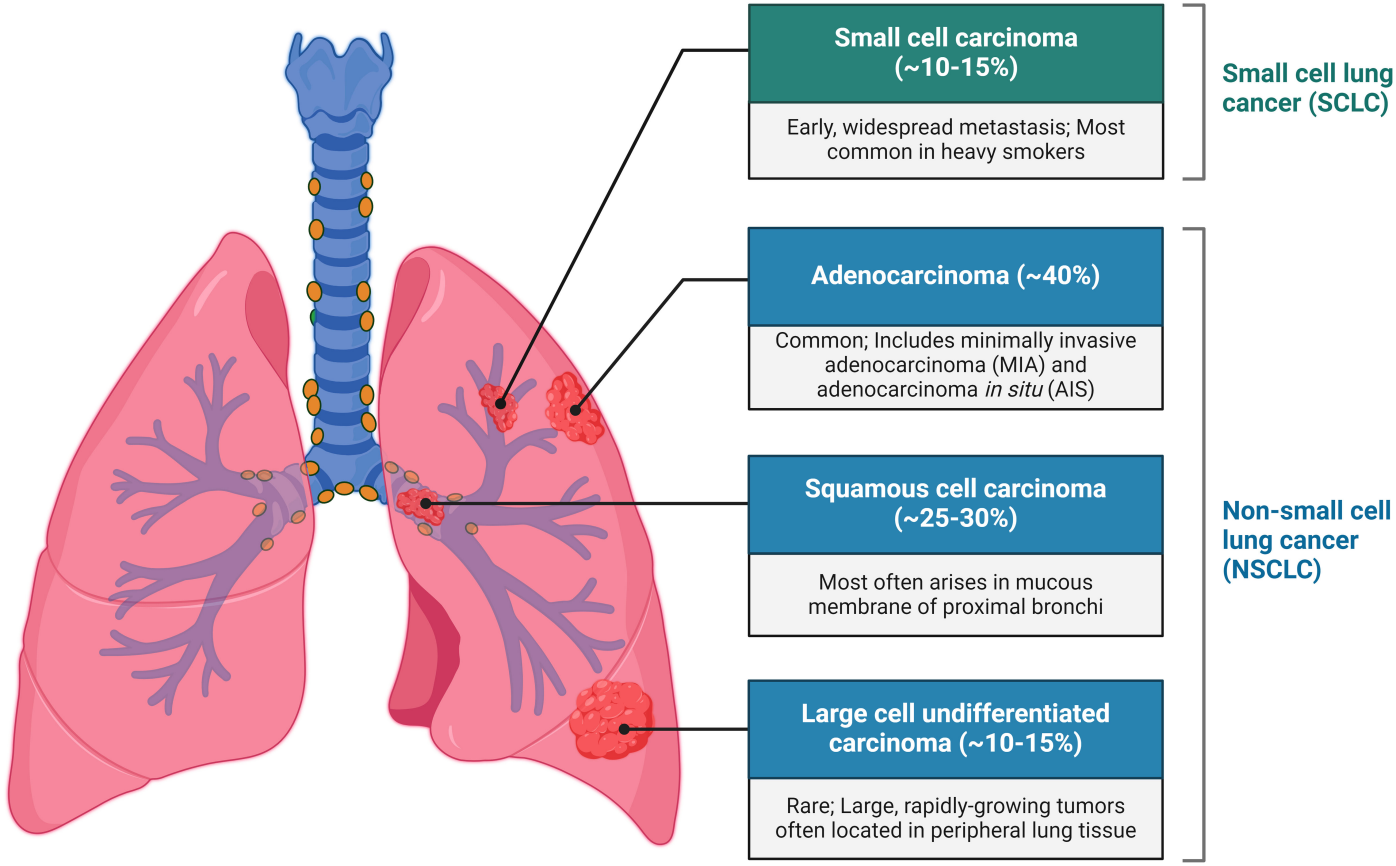
Types of lungs cancer.

Lung cancer is the most common and leading cause of cancer-related deaths worldwide and primarily comprises two subtypes: small-cell lung carcinoma (SCLC), accounting for 15%, and non-small cell lung carcinoma (NSCLC), accounting for 85% (1). NSCLC is further categorised into three main types: adenocarcinoma, squamous cell carcinoma, and large cell undifferentiated carcinoma (**Figure 1**) (2). Smoking is significantly associated with SCC and LCC, while adenocarcinoma is the most prevalent type, constituting ~40% of cases among both smokers and non-smokers. The overall 5-year survival rate for lung cancer is slightly above 16% (3), with more than 50% of cases being diagnosed at a late stage. Early diagnosis greatly improves prognosis; for instance, stage 1A1 has a 5-year relative survival rate of 90%, whereas survival drops below 10% by stage 4 NSCLC (4). The common methods of treatment for cancer are targeted therapy and immunotherapy such as immune checkpoint inhibitors (ICIs) and tyrosine kinase inhibitors (TKIs), the PD-1/PD-L1 axis, as well as cytotoxic T-lymphocyte antigen-4 (CTLA-4) (1). The treatments mentioned although frequently used have had unimpressive outcomes accompanied by risk of infections (5). Thus, the ongoing research on the use of cancer biomarkers as diagnostic or prognostic tools for various types of cancer is necessary since cancer cells undergo genetic or epigenetic modification resulting in alterations of a protein, metabolite, nucleic acid, or hormone (6).

For the past few years, studies have begun to highlight exosomes, which are tiny extracellular vesicles (30–120 nm) that originate from endosomes, which can be found in bodily fluids such blood, urine, saliva, and amniotic fluid (7). The biochemical composition of exosomes consists of lipids and proteins nucleic acids, primarily mRNA and miRNA as well as mitochondrial and genomic DNA (8). There are numerous characteristics that make exosome useful for diagnosis and monitoring for instance tumour exosomes can be used for liquid biopsy as they are enriched with biofluids. The differences in the expression of biologically active substances in exosomes between healthy individuals and cancer patients can enhance the specificity and sensitivity of early tumour diagnosis (9). Moreover, the promising gene and medication delivery vehicles due to relative stability, robustness, immunomodulatory and regenerative qualities of exosomes are remarkably essential features as a disease biomarker. Studies done by Fraser (2016) discovered that leucine-rich repeat kinase 2 (LRRK2) is a biomarker in Parkinson Disease patients’ urine exosomes that indicates the likelihood that those who carry the LRRK2 mutation will develop the disease and that α-synuclein aggregation could be a significant factor in Parkinson’s disease pathogenesis (10). Alzheimer’s disease (AD) is another neurodegenerative illness in which early diagnosis has been achieved via early identification of exosome-associated tau, such as phosphorylated at Thr-181 (AT270) and found in human cerebrospinal fluid (CSF) samples (11). Lastly exosome-derived miRNA or exosomal miRNA have been linked to medication resistance in a variety of malignancies, according to mounting evidence such as Tamoxifen resistance which has been observed to rise due to breast cancer exosome-derived miRNA-221/222 (12).

On the other hand, tumour-derived exosomes in lung cancer can limit the growth of immune cells, trigger the death of activated CD8+ T effector cells, lessen the activity of natural killer cells, prevent monocyte differentiation, and encourage the growth of regulatory and myeloid-derived suppressor T cells. In addition, the theragnostic application of exosomes in lung cancer for instance, cisplatin-loaded exosomes can be used to treat hepatocellular carcinoma (13). In cancer patients, especially those with NSCLCs, biomarkers such as the expression level of miR-155 play a predictive function. Research on drug delivery using exosomes derived from macrophages and loaded with paclitaxel has shown that these macrophage-derived exosomes are more effective in inhibiting cancer cell growth compared to earlier drug-loading methods (14). Exosomes were recently extracted from NSCLC patients’ blood using an Extracellular Vesicles (EV) array, and scientists used these exosomes to build a diagnostic model with 30 exosomal proteins that had a sensitivity and specificity of almost 75%. Therefore, EV Arrays have the ability to diagnose lung cancer in addition to determining the proteome profile of exosomes from tumour cells thus patients can be classified as either malignant or noncancerous based on a number of proteins found in exosomes (15). Additionally, CD151, CD171, and tetraspanin 8 were important markers to distinguish patients with all histology lung cancer from cancer-free individuals thus highlighting promising use of exosomes in lung cancer diagnosis (9). Above evidences demonstrates that numerous studies are currently being carried out to develop strategies for utilizing exosomes as efficient carriers for therapeutics delivery, as well as to explore novel approaches for diagnosing and anticipating outcomes using LCCDEs. The current study’s goal is to give comprehensive reviews of the state of lung cancer at present-day and its diverse applications of LCCDEs. Comprehensive diagnostic and therapeutic strategies employing LCCDEs have also been presented, along with information on the findings of the most current exosome research clinical studies and their current status. The promising results and technologies indicate that the clinical treatment of lung cancer patients could benefit from the use of LCCDEs.

## 2 Exosome and its biological properties and functions

### 2.1 Structure of exosome

**Figure 2:**
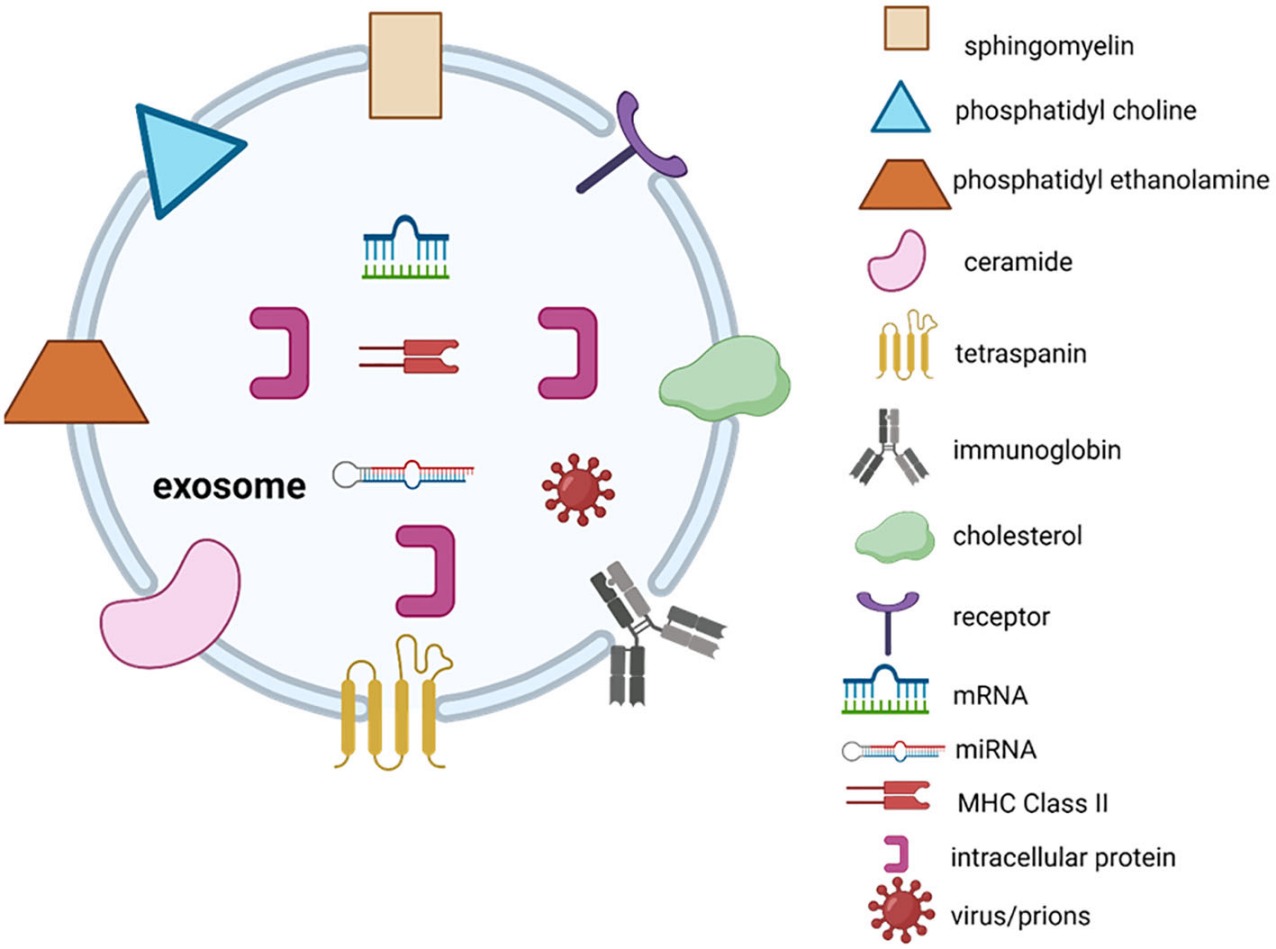
Exosome structure.

Exosomes have a significant impact on the initiation and growth of tumours in cancer. Furthermore, they play a crucial role in the invasive advancement of cancer through mechanisms such as suppressing the immune system by inhibiting the growth of immune cells, causing apoptosis of activated CD8+ T cells, and suppressing the function of natural killer cells (16). Exosomes typically contain membrane transport as well as fusion proteins like Rab GTPases, annexins, flotillins, micro-vesicle body biogenesis genes and proteins like Alix and tumour susceptibility gene Tsg101 (17). Moreover, the protein groups linked to lipid microdomains, such as integrins and tetraspanins (e.g., CD9, CD81, CD82, CD83, and CD63), play a crucial role in regulating exosomes (18). The pool of commonly encountered proteins includes β-actin, tubulins, myosin, cofilin, glyceraldehyde 3-phosphate dehydrogenase, and the major histocompatibility complex, also known as MHC, class I and II molecules, which are associated with the cytoskeleton and metabolism (19). Antigen presenting cells, such as B lymphocytes and dendritic cells (DC), release exosomes that carry MHC class-I and class-II molecules. These exosomes have the ability to promote the growth of T cells in laboratory settings (15). The wide range of proteins secreted by exosomes, many of which play important roles in influencing various signaling pathways, has led to extensive research on exosomes in cell-to-cell communication. Exosomes are believed to have the ability to control the activity of distant cells by releasing their contents at a considerable distance from where they originated. This can potentially impact the functions of the recipient cells, particularly by facilitating communication between different cell types in the tumour microenvironment. For instance, the transfer of RNA from one cell to another, referred to as “exosomal shuttle RNA,” has the potential to impact protein synthesis in the receiving cell (20). Exosomes derived from specific immune cells, such as DC and B cells, have the ability to transfer molecules between cells. These exosomes are believed to have a functional role in facilitating adaptive immune responses against pathogens and malignancies (21). On the other hand, the production and composition of exosomes can be affected by molecular signals that are received by the cell from which they originate (22). Microparticles are a type of exporters that are small vesicles enclosed by a protective membrane. They are found in the circulation of blood and originate from cells that come into contact with the circulatory system, such as platelets and endothelial cells (23). **Figure 2** describes the structure of exosomes. In this illustration, exosomes have the ability to contain a diverse range of molecules, such as viruses, nucleic acid material (RNAs, DNA), and microRNAs. The specific molecules present in exosomes depend on various factors, such as the type of cell or its origin. Additional factors that affect the content of exosomes include the pathological condition of the living organism. Exosome contents can be transferred from the cell they originated from to target cells in the surrounding microenvironment or even at afar, potentially leading to the formation of extensive intercellular communication networks.

### 2.2 Biogenesis of exosome

**Figure 3:**
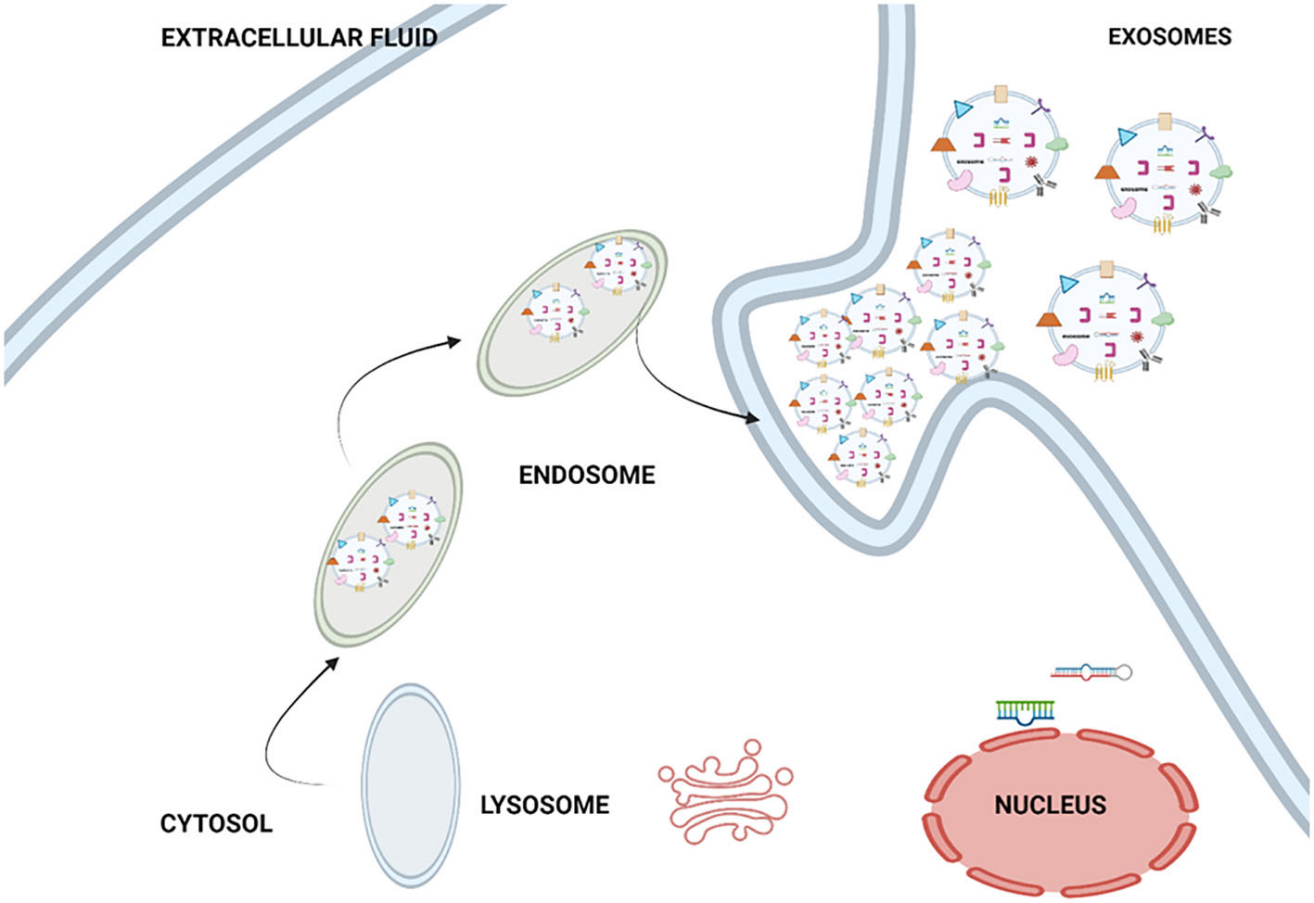
Biogenesis of exosome.

Endocytic pathways involved in the formation of exosomes. The process of exosome biogenesis involves several distinct stages, namely the formation of early endosomes, the formation of late endosomes, and the formation of multivesicular bodies (MVBs) (24). Afterwards, the MVB is either transported to the lysosome for lysosomal exocytosis or merged with the membrane of the endosomal cells, resulting in the discharge of exosomes into the extracellular space (24). The Golgi apparatus and endoplasmic reticulum are the two major organelles depicted, as they communicate with early endosomes immediately after they develop from endocytic vesicles (24). **Figure 3** shows the biogenesis of the exosome. This figure illustrating the widely accepted model for the mechanism of exosome formation and release. Exosomes originate mainly from multi-vesicular bodies (MVBs), and these are late endosomes that first evolved from lysosomes. Exosome production can be initiated by various factors, such as external stimuli (e.g., microbial invasion) and other forms of stress. Exosomes are able to be discharged into the extracellular milieu through the merging of MVB with the outer surface of the cell, a process facilitated by a set of specific proteins known as soluble N-ethylmaleimide-sensitive fusion attachment protein receptor (SNARE) (25).

EVs are released by nearly all kinds of cells and are primarily categorised as exosomes, MVs, and apoptotic bodies based on their shape, dimensions, and formation origin (26, 27). Different EVs merge to form an endosome, which subsequently matures into MVB. MVBs form when the outermost membrane of endosomes folds inward, creating intraluminal vesicles (ILVs) that contain cytosolic substance (28). The nominal diameter of ILVs ranges from 48 to 95 nm. The synthesised MVBs originate from two primary sources, namely, the intermediate stage of intracellular protein degradation or the process of exosome synthesis (29). While the precise mechanisms governing the outcome of an MVB are not fully comprehended, certain investigations indicate that the ultimate fate of a specific MVB is contingent upon the quantity of cholesterol it contains. Therefore, the individual with a greater cholesterol level is chosen for subsequent secretions, while the others (with a low cholesterol concentration) are directed towards lysosomal breakdown (30, 31). In contrast to the production of other MVBs, which primarily occurs through the process of “budding off” from the outer layer of cells, the process of biosynthesis of exosomes is considerably more intricate. The process involves the inward folding and subsequent release of MVBs, resulting in the secretion of exosomes into the extracellular fluid (32). Three essential processes are involved in the transportation and secretion of MVBs outside of the cell to generate exosomes: MVBs are targeted for transport, attach of the MVBs to the plasma membrane, and the MVB restriction membrane fuses with the plasma membrane. The MVB surface proteins are necessary for this mechanism to function efficiently. The receptor on the target membrane selectively recognises and binds these proteins and the entire process acts as a conveyor belt to move the MVB to its intended spot. Numerous investigations have demonstrated the involvement of the SNARE protein family, cytoskeletal proteins, tethering factors, and the RAB GTPase protein family in these regulatory mechanisms. The process of MVB docking and fusing with the plasma membrane is mediated by SNARE proteins, whilst cytoskeletal proteins offer dynamic assistance during transport. The subcellular locations of the many proteins in the RAB family vary within the cell. In order to guarantee the precision of MVB membrane transport and membrane fusion, they not only mediate transport to the plasma membrane but also oversee all of the previously mentioned procedures (33). Exosomes are thought to be released at the same time that plasma membrane-fused MVBs are being secreted, according to conventional wisdom (27). Moreover, the cargo of exosomes acts as a reliable indicator of the molecular processing that occurs within the parent cell. It also has the potential to act as a substitute for the parental cell in body fluids (32). In addition, the MVBs and their cargo protein casing interact with the endosomal sorting complex required for transport (ESCRT), specifically ESCRT-0, 1, 2, and 3, which are associated with various proteins such as Vps4, Flotillin, Tsg101, and Alix. The budding membrane is formed through the association of the ESCRT-0 complex with ESCRT complexes 1 and 2 following membrane deformation. Collectively, these complexes combine with ESCRT-3 and Vps4 protein, resulting in the creation of ILVs by cutting the narrow connection of the protrusions (34, 35). There are alternative pathways, independent of ESCRT, for the formation of exosomes. These pathways involve the utilization of lipid microdomains associated with rafts on the cell membrane, as well as specific proteins such as tetraspanins. Sphingomyelinases enhance the lipid rafts and catalyse the conversion of sphingomyelin into ceramide. This conversion triggers a spontaneous formation of ILVs through membrane budding (36). The precise role of tetraspanin proteins in the process of vesicle formation remains incompletely comprehended. The tetraspanin-enriched microdomains (TEMs) are involved in various processes such as the transportation of cargo molecules, the clustering of specific receptors, and the processing of important molecular components into exosomes (37).

### 2.3 Isolation of exosome

The present understanding of the biological functions of exosomes has primarily depended on several techniques for isolating EVs. Hence, it is crucial to possess the ability to promptly and accurately distinguish exosomes from various forms of cellular waste and other EVs. Different isolation procedures can be employed to separate exosomes from biofluids or cell culture supernatant, based on their size and affinity. Contrary to the methods employed for separating nucleic acids and proteins, the methods for isolating exosomes have been established relatively recently, during the last few decades. The similarity in size between exosomes and other EVs, such as ectosomes and MVs, has significantly hindered the progress of isolation methods. Over the past few decades, there has been a growing exploration of various methods for isolating exosomes. The key mechanism of these approaches allows for their broad classification. Methods such as UC (ultracentrifugation), density gradient (DG) centrifugation, infiltration techniques, immunoaffinity, capture-based approaches, exosome precipitation, and the utilization of acoustic nanofilters are employed (20, 38).

UC is the predominant method for isolating exosomes and is essential in the exosome isolation process. DG centrifugation, a modified version of ultracentrifugation, is widely regarded as the most reliable method for isolating exosomes and is considered the benchmark for this purpose (39). By subjecting the sample to rapid centrifugation using a series of specific parameters, it is possible to effectively exclude dead cells, cellular debris, and apoptotic bodies. This process allows for the separation of a diverse array of exosomes based on their ability to form pellets. Traditional UC is commonly employed due to its efficacy in analysing various biofluids, such as serum, urine, cerebrospinal fluid, breast milk, aqueous humour, and amniotic fluid (40). Prior to 2015, UC emerged as the predominant approach for isolating exosomes from cell culture supernatant and biological fluids throughout the advancement of novel exosome separation techniques (41). Nevertheless, the productivity and quality of exosomes obtained during ultracentrifugation are significantly influenced by various factors, such as the type of rotor, duration of centrifugation, and viscosity of the material (42). Similarly, it is important to take into account these characteristics when utilizing and enhancing UC methods for certain sample types. Differential gradient centrifugation is a well-established method for separating subcellular components based on their buoyant density, leading to enhanced particle separation efficiency (43). Differential gradient centrifugation is employed to segregate exosomes based on variations in their size and density compared to other constituents, necessitating distinct centrifugal strengths and durations for sedimentation. Differential gradient centrifugation has been widely employed to process a diverse range of samples, such as plasma, cell culture supernatant, serum, saliva, urine, and breast milk. One method that has been employed to isolate extracellular vesicles, such as exosomes, from salivary fluid is DG centrifugation. Salivary fluid contains a combination of gland secretions, gingival crevicular fluids, cell debris, and bacteria. While this technique is simple to execute and produces exosomes of superior quality, it is a time-consuming procedure that heavily relies on specific instruments. Recent research has found that repeated umbilical cord procedures result in exosomes with low productivity and negative impacts on the quality of exosomes. These effects make the exosomes unsuitable for use in therapeutic applications. Moreover, it has been documented that this technique can produce exosomes that may be impaired, presumably as a result of the intense shear forces exerted on the exosomes during high-speed centrifugation (44, 45).

Size-based techniques can be broadly classified into three primary categories: sequential filtration, ultrafiltration, and size exclusion chromatography (SEC). Ultrafiltration, which employs a molecular weight cutoff (MWCO) ranging from 10 to 100 kDa, is frequently utilised as an initial stage in the concentration of exosomes from substantial volumes of source material into minute-volume samples suitable for subsequent purification processes (46). The procedure of sequential filtration is commonly categorised into three distinct stages. The initial stage involves the filtration of cells and cellular debris, followed by the depletion of free proteins and concentration of the samples. Ultimately, exosomes are sorted using filters that have precise and predetermined pore diameters (47). SEC, in comparison to centrifugal and filtration techniques, has several benefits such as repeatability, cost-effectiveness, and non-destructive results. Crucially, this technology can also be used to collect exosomes from serum and plasma. A sophisticated method called sequential centrifugal ultrafiltration (SCUF) has been employed to isolate exosomes with a high level of purity and to separate out MVs from a human colon cancer cell line (48). A recent study has demonstrated that ultrafiltration is a superior option to UC due to its ability to achieve the maximum retrieval rate of particles smaller than 100 nm, including exosomes. The use of NanoSight and transmission electron microscopy (TEM) revealed that the size distributions of exosomes obtained using ultracentrifugation or size exclusion chromatography (SEC) were comparable. Ultrafiltration techniques offer a greater particle yield compared to the standard UC approach, resulting in increased exosome yield and isolation efficiency in a shorter amount of time. Although size-based approaches have found extensive application across various domains, their practicality in treatment and research is hindered by their somewhat lengthy execution time.

Capture-based methods, which are intimately associated with immunoaffinity, are frequently employed to generate exosomes of exceptional purity (49). Magnetic beads, a unique tool that may be manipulated to attach to specific proteins on membrane surfaces, are crucial in capture-based approaches. Exosomes possess several membrane proteins, including CD9, CD63, ALIX, and Ep-CAM, which can be concentrated using magnetic beads covered with antibodies (50). The immobilization of certain exosomes can be effectively accomplished by washing them in a stationary phase, depending on the precise immunological interaction between the antibody and antigen. This approach effectively fulfils the stringent requirements of isolating exosomes that possess certain target membrane proteins. The consensus about the superiority of capture-based strategies utilizing the Ep-CAM biomarker for exosome separation, as opposed to alternative methods, has been generally acknowledged based on thorough evaluations of exosome recycling efficiency (51). A recent study has shown that the use of the Vn96-peptide to isolate extracellular vesicles from urine, which selectively binds to EVs harbouring a heat shock protein, is significantly quicker than conventional approaches like ultracentrifugation in the context of prostate cancer (52). Although the exact mechanism of this heat shock-based isolation methodology remains uncertain, it undeniably promotes the advancement of sophisticated methodologies that are beneficial not only for prostate cancer but also for other malignant tumours. The IAC-Exo system, which utilises targeted immunoaffinity and magnetic bead capture methods, is the most effective technique for enriching exosomes when compared to DG centrifugation and UC. Due to its ability to capture a greater quantity of exosomes compared to the other two approaches, immunoaffinity-based isolation of exosomes (IAC-Exo) has been suggested for widespread application in sectors related to exosome-based treatment and research. The exoRNeasy Serum/Plasma Kit, developed by Qiagen in Hilden, Germany, utilises a membrane-based affinity binding approach to effectively purify total exosome-derived RNA from serum and plasma samples. Hence, this commercial kit unquestionably embodies a system that utilises capture-based methodologies (53, 54). By utilizing immunoaffinity isolation, this approach enables the segregation of unique exosome subpopulations generated by specific cell types, facilitating the examination of variations in the functional impacts of these exosome subpopulations. Moreover, this approach enables the imaging of individual exosomes and the identification of protein markers on individual exosomes. Regrettably, magnetic bead-based separation techniques are unsuitable for exosome separation on a wide scale. Furthermore, the exorbitant expenses and meager productivity impede their progress and utilization.

Contrary to the aforementioned isolation approaches, precipitation techniques primarily rely on the utilization of polymers to cause the precipitation of exosomes, which are subsequently processed for subsequent purification. Polyethylene glycol (PEG), a widely utilised polymer in exosome isolation, significantly enhances the concentration and quantity of exosomes (55). Prior to its application with exosomes, this technique was documented as viable for separating diverse biomolecules and viruses from physiological fluids (56). In this procedure, the samples are incubated along with a PEG solution at a temperature of 4 °C for the duration of one night. Following this period of incubation, a sequence of separation techniques, such as filtration and centrifugation, can be employed to continue the processing of the precipitate containing exosomes. Due to the increasing need for improved effectiveness and efficiency in exosome isolation procedures, biotech companies are increasingly focusing on creating commercial products for exosome isolation. Some notable examples include ExoQuick by System Biosciences in the United States, Total Exosome Isolation Reagent by Invitrogen in the United States, ExoPrep by HansaBioMed in Estonia, Exosome Purification Kit by Norgen Biotek in Canada, and miRCURY Exosome Isolation Kit by Exiqon in Denmark. Nevertheless, the efficacy and quality of commercial exosome isolation kits differ. Research has shown that when compared to two other polymer-based kits (ExoQuick™ or OptiPrep™), the Exo-spin™ kit is the most effective commercial method for extracting exosomes. This is because it produces a higher quality and purer yield (57). Contemporary precipitation techniques are appealing for clinical use due to their little requirement of initial material when dealing with human biofluids and their compatibility with high-throughput alternatives.

**Figure 4:**
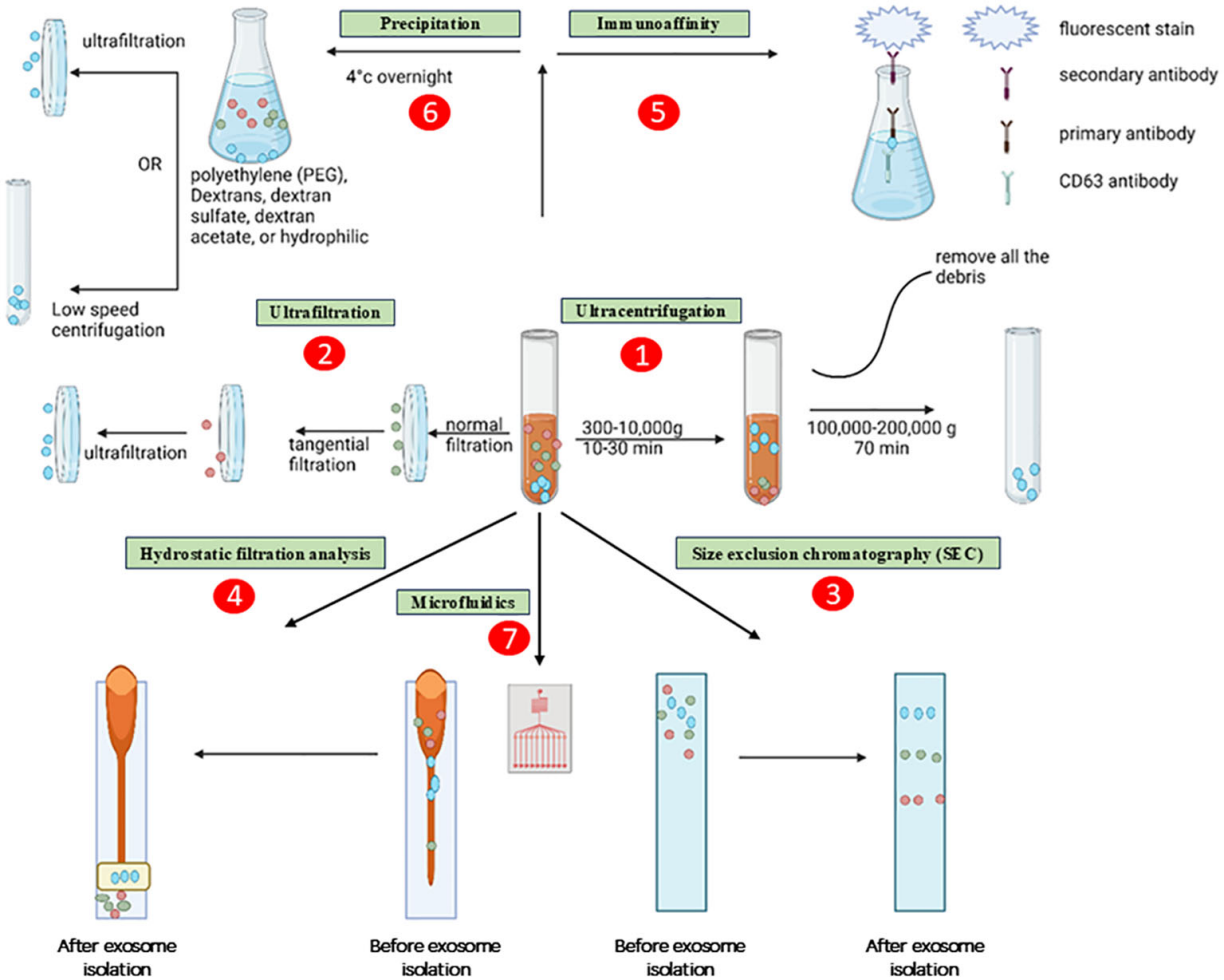
Methods for isolating the exosomes. The exosomes are depicted as little, ebony spheres. The procedures are delineated as several paths, namely: (1) ultracentrifugation, (2) ultrafiltration, (3) size exclusion chromatography, (4) hydrostatic filtration dialysis, (5) immunoaffinity, (6) precipitation, and (7) microfluidics.

Microfluidics devices are very suitable for the separation of exosomes from other particles of nanoscale size due to their ability to provide cost-effective, rapid, and accurate isolation procedures (58). Microfluidics-based approaches are recognised for their distinctive characteristics, such as cost-effectiveness and time efficiency. Furthermore, these solutions address a fundamental issue by circumventing the discontinuous separation procedures associated with alternative methods. Presently, commonly employed microfluidics technologies are completely integrated with size-based separation, immunoaffinity-based separation, and dynamic separation. The ExoTIC device, a novel exosome isolation technology, has been launched in recent years. The ExoTIC gadget gained popularity throughout time because to its unquestionable benefits, such as its high productivity, purity, and efficiency. Compared to PEG precipitation (including the ExoQuick™ approach) and UC, the ExoTIC device is more suited for extracting exosomes from serum or other physiological fluids (59). Although it possesses several benefits such as exceptional purity, precise control, specific isolation, and remarkable efficiency, there are still certain challenges associated with it. These challenges include the necessity for intricate separation devices and restrictions arising from the demand for high immunoaffinity (60). Microfluidic platforms have been extensively developed not just for exosome isolation, but also for the separation of DNA, proteins, and viruses. Despite the presence of numerous anticipated obstacles, there is a strong inclination to investigate the application of microfluidics-based methods for widespread utilization in processes specifically targeting the extraction of diverse bioactive substances, such as exosomes (61, 62). Primarily, an optimal approach for exosome isolation should possess characteristics such as simplicity, speed, efficacy, affordability, and scalability. Furthermore, it should not cause harm to the exosomes or necessitate the use of extra equipment. Different approaches possess distinct advantages and disadvantages for efficiency, reproducibility, and impact on functional outcomes. To address these drawbacks and expedite exosome research in both fundamental and medical contexts, it is imperative to enhance isolation protocols and employ a combination of isolation strategies. **Figure 4** shows the various methods for isolating the exosomes.

### 2.4 Detection of exosome

The correct identification and detection of exosomes is becoming increasingly important in clinical research due to their many important uses in health science and medicine. Biomolecules that can be recognised, including lipids, proteins, and nucleic acids, are utilised by a variety of detection methods (63). Biomarkers that facilitate the identification of exosomes include a variety of lipids, protein molecules, and nucleic acids that are involved in exosome synthesis and release. Optical, electrochemical, immunoreaction, fluorescence, surface plasma resonance (SPR), surface-Enhanced Raman Scattering (SERS), chromatographic, and microfluidic detection methods are among the most used approaches to identify exosomes. The total procedure is now more efficient thanks to the combination of one or more detection methods (64–66).

#### 2.4.1 Nucleic acid-based detection of exosomes

The RNA, DNA, and microRNA that make up nucleic acids reflect crucial genetic information regarding the genesis, function, physiological alterations, and destiny of preexisting exosomes (67). The ability of these nucleic acid compounds to freely communicate and carry information between the body’s cells is often associated with diseases, especially when it comes to the detection of malignancies. This opens the door to potential uses in cancer medication therapy based on RNA (68). The detection of nucleic acids is usually accomplished by next-generation sequencing or electrochemical techniques. Since most genetic materials (DNA/RNA) are situated inside the exosome, they are still encased in the lipid bilayer, which makes nucleic acid detection difficult to accomplish during diagnostic procedures. A workaround for this is the release and subsequent identification of nucleic acids. Research by Zhang et al. (69) used an adapter magnetic bead bioconjugate to stimulate LNCaP cell release of several mitochondrial DNAs (mDNAs). A gold electrode served as a hybridization platform for the released mDNAs and the probing DNAs. Electrochemical signals revealed the presence of tumour-associated exosomes through these mDNAs. Another study focused on analysing DNA nanostructure and nano-tetrahedron to develop an aptamer as electrochemiluminescence (ECL) for identifying malignant exosomes from hepatocyte cells (70). According to Wang S (2017) (70), the aptasensor system’s maximum limit of detection was found to be 3.96 × 10¹/mL. Another work that looked at next-generation sequencing for nucleic acid identification was Sun et al. (71). The miRNA expression profile in bovine milk was studied using next-generation sequencing. Bacterial infection was suggested by an elevation in the level of exosomal miRNA, which highlights the potential for early detection of bacterial infection in the mammary gland.

#### 2.4.2 Protein-based detection of exosomes

According to the existing literature, exosomes are highly effective diagnostic indicators due to the presence of certain proteins on their surface that distinguish them from other vesicles (72). One option is to use a single protein type to specifically collect and identify exosomes; another is to use numerous proteins for exosome analysis. The abundance of tetraspanins on the exosome surface makes them ideal biomarkers for quantitative exosome analysis, among the many proteins detected on the exosome surface. In a similar vein, urinary exosomes containing biomarkers (such as aquaporin-1) and the exosomal protein CD24 are linked to the rapid diagnosis of autoimmune disorders and kidney damage, respectively (73, 74). Immunoreactions, aptamers, and surface plasmon resonance-based techniques are frequently used for protein identification.

#### 2.4.3 Lipid-based detection of exosomes

The lipid bilayer that encases exosomes provides them with inclusion stability and is abundant in components such as cholesterol, phospholipids, and polyglycerol. Consequently, several methods have been devised to specifically target these lipid components in order to detect exosomes using an aptamer-based strategy. As a result, interference signals have been significantly reduced, and the exosome detection system is highly sensitive and selective. Double marker recognition occurs when, in addition to the usual exosome recognition and detection, lipids occasionally bind to the surface protein on the exosomes. As an example, a strategy that improves the method’s sensitivity by a factor of five uses cholesterol as a probe for target binding with aptamers or surface proteins such as CD63, which are occasionally coupled with a magnetic separation technique (75). Skotland et al. (2017) conducted a study where they identified and separated different lipid compounds found in exosomes from urine samples of patients afflicted with prostate cancer (76). In addition, the measurement of various lipids was conducted utilizing techniques such as mass spectrometry (MS) and lipidomics. The phospholipid levels were elevated in PC-3 cells. This demonstrates the diagnostic importance of exosomal lipids. Zhang et al. (2019) conducted a study where they created a DNA probe modified with cholesterol to specifically connect with CD63 aptamer (77). They used magnetic beads to separate and capture exosomes. The strategy was determined to be straightforward and uncomplicated to execute. In order to enhance the sensitivity and magnification of signals, we conducted a hybrid chain reaction (HCR) using alkaline phosphatase. This allowed us to quantitatively examine exosomes either through visual identification or by using a UV-vis spectrophotometer. The limit of detection (LOD) for the new detection technique was determined to be 1.6 × 102 particles/mL, demonstrating its efficiency and reliability as an exosomal detection and quantification system (77).

## 3 Tumour-derived exosomes

**Figure 5:**
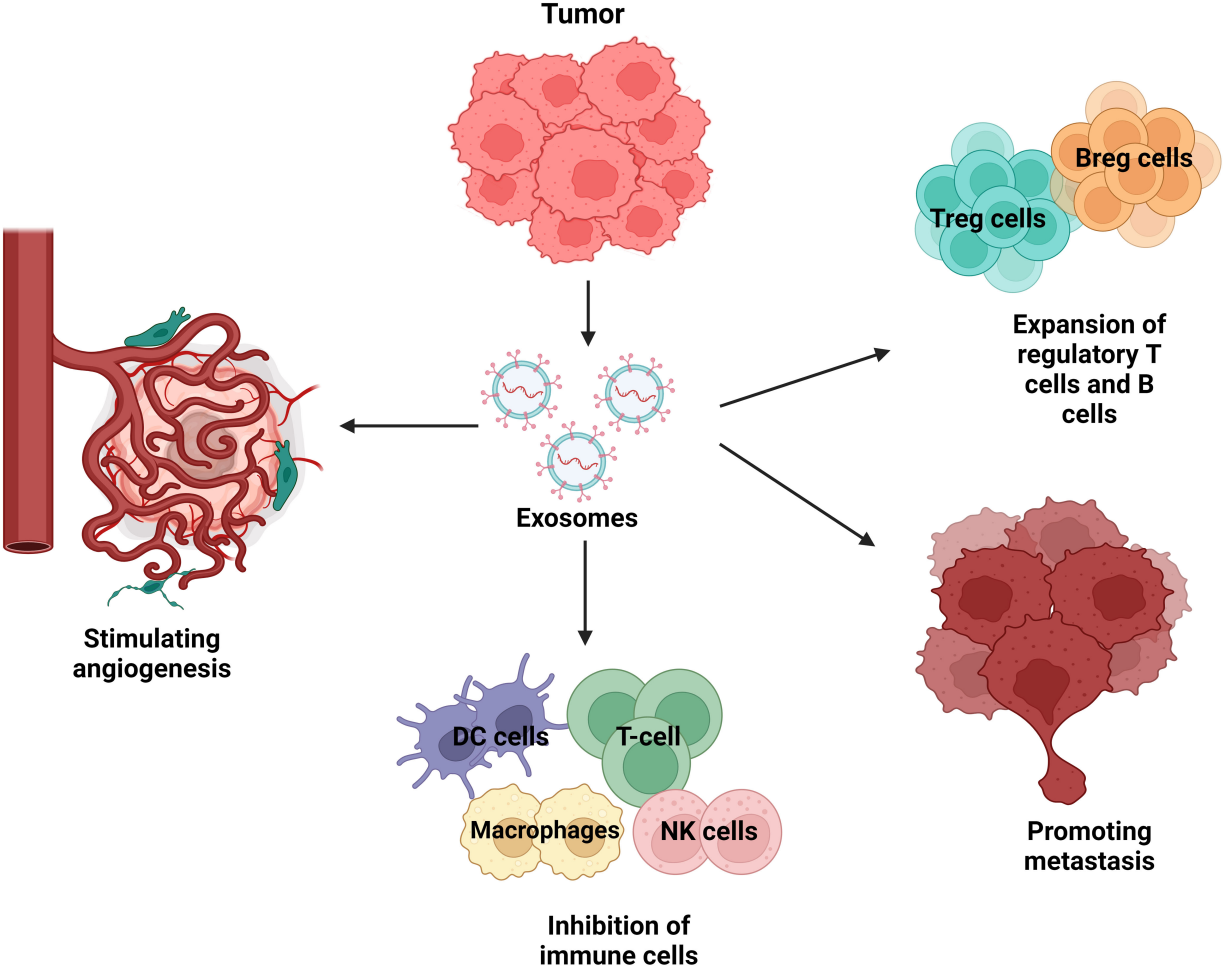
Tumour-derived exosomes (TDEs) in the tumour microenvironment. Exosomes from tumour cells abundantly release exosome to send signals to neighbouring cells. Recipient cells such as surrounding cells, tumour cells, endothelial cells, and immune cells are functionally affected by tumour-derived exosomes. TDEs regulate a variety of cellular mechanisms that promote tumour growth, immune evasion, angiogenesis, and metastasis. TDEs block immune cells such as T cells, natural killer (NK) cells, dendritic cells, and macrophages, limiting the immune system’s ability to combat tumours. This immune suppression allows the tumour to elude detection and elimination. TDEs also increase the proliferation of regulatory T cells (Tregs) and B cells (Bregs), which block anti-tumour immune responses, hence boosting the tumour’s survival. TDEs also have an effect on endothelial cells via stimulating angiogenesis, which is the development of new blood vessels. This mechanism provides nutrients and oxygen to the tumour while also increasing blood vessel permeability, which promotes metastases. Lastly, TDEs contribute to metastasis by preparing distant tissues for the entrance of circulating tumour cells, which aids in the spread of cancer to other organs.

TDEs play a role in tumour spreading and are actively created and released by tumour cells. They convey instructions from tumour cells to aberrant or healthy cells. The tumour microenvironment contains a substantial amount of tumour-derived exosomes (TDEs), which play a number of roles in promoting tumour metastasis (78). These mechanisms include the acquisition of primary tumour migration capacity, tumour angiogenesis, immune system organotrophic metastasis escape, formation of the pre-metastatic niche, and metastatic tumour growth in the secondary site (79). TDEs stimulate the proliferation and activation of regulatory T cells and B cells, while suppressing the activity of effector T cells and NK cells, resulting in the development of immunosuppressive tumour microenvironment (80). TDEs promote tumour growth and metastasis by not only converting normal fibroblasts into cancer-associated fibroblasts (CAFs), but also by stimulating the transformation of epithelial cells into a mesenchymal phenotype. Tumour angiogenesis is facilitated by TDEs, which stimulate the activation of endothelial cells (81). For example, the TDE of non-small cell lung cancer (NSCLC) has been shown to contain pro-angiogenic factors such as vascular endothelial growth factor (VEGF) and basal fibroblast growth factor (bFGF) while Time released these substances into the tumour microenvironment, these substances can stimulate endothelial cell activation and induce new blood vessel formation (82). These new vessels not only provide the tumour with essential nutrients and oxygen, but also promote the spread of cancer cells to distant sites through the bloodstream, leading to metastasis Thus, TDE-mediated angiogenesis plays an important role in lung cancer progression reveal future therapeutic importance (83). **Figure 5** shows the involvement of TDEs in the tumour microenvironment and how they can promote cancer development.

TDEs can induce a localised signaling response at the location of metastasis. Previous study, suggesting that TDEs could stimulate the creation of pre-metastatic niches, hence promoting the advancement of cancer metastasis (84). Several research have presented evidence indicating that TDEs can impede the immune response against specific tumour cells, leading to the evasion of the tumour. For instance, TDEs released by breast cancer cells contain immunosuppressive substances that suppress immune cell function and enhance the immunosuppressive tumour microenvironment, and cause the tumour evades immune surveillance (85). Moreover, TDEs have been identified as a significant contributor to the development of drug resistance (86). Research has demonstrated that exosomes derived from colorectal cancer cells have the ability to stimulate hepatic stellate cells to release IL-6 (87). IL-6 release has been demonstrated to control the lactate metabolism of tumour cells that are deprived of oxygen, hence providing resistance to SN38, the active form of irinotecan. In addition, cisplatin-resistant NSCLC cells transfer drug resistance to sensitive cells by enhancing glycolysis through exosome-mediated delivery of PKM2 (88).

Apart from that, TDEs can facilitate the evasion of the immune system and the monitoring of immune responses by impeding the survival and activation of lymphocytes. For instance, exosomal miR-203 derived from pancreatic cancer cells can be taken up by DCs, which are crucial antigen-presenting cells (APCs) (89). Eventually, exosomal miR-203 leads to dysfunction of DCs by suppressing the expression of TLR4, TNF-α, and IL-12. Furthermore, exosomal miR-212–3p produced from pancreatic cancer (PaCa) cells can hinder the ability of DCs to present antigens to T lymphocytes. This inhibition is achieved by suppressing the production of transcription factors specific to major histocompatibility complex II (MHC II) and regulatory factor x-associated protein (RFXAP). Furthermore, exosomes generated from PaCa cells suppress the proliferation of T-cells via regulating the production of IL-12. Signals facilitated by TDE can impede the functioning of immune cells at various stages (90).

Drug resistance poses a significant challenge and serves as a primary constraint for targeted therapy. Increasing data indicates that TDEs have a role in developing resistance to EGFR-TKIs by transferring active substances, specifically exosomal miRNAs (91). According to the findings, the exosomes generated by EGFR-TKI-resistant H827R cells caused a reduction in the sensitivity of NSCLC HCC827 cells to gefitinib (92). Furthermore, the suppression of miR-21 eliminated the drug resistance caused by exosomes in HCC827 cells, which is in line with the previously documented function of miR-21 in NSCLC cells that are resistant to EGFR-TKI treatment (82). TDEs additionally promoted the ability of drug-resistant tumour cells to evade apoptosis. The levels of exosome-secreted long non-coding RNA AFAP1-AS1 were considerably higher in trastuzumab-resistant cells that showed resistance to apoptosis, compared to drug-sensitive cells. The overexpression of AFAP1-AS1, induced by the alteration of H3K27ac, promotes the translation of ERBB2 by binding to AU-rich binding factor 1 (AUF1), an mRNA decay factor. This ultimately results in the development of resistance to trastuzumab in breast cancer (83).

Angiogenesis is a complex series of steps that allows tumours to create new blood vessels, which is crucial for their growth and spread throughout the body (93). The production of exosomes by tumour cells is a primary mechanism that stimulates the development of blood vessels. VEGF, fibroblast growth factor (FGF), Platelet-derived growth factor (PDGF), basic fibroblast growth factor (bFGF), transforming growth factor β (TGF-β), tumour necrosis factor α (TNF-α), and interleukin-8 (IL-8) are prominent angiogenic stimulatory factors that are transported by TDEs (94). The growth of a tumour relies on its ability to access the host vasculature, which allows the tumour to connect with the bloodstream. In order to accomplish this, tumours induce the formation of their own network of blood vessels by altering the tumour microenvironment (TME) from an anti-angiogenic state to a pro-angiogenic state, a process known as the “angiogenic switch”. TDEs have been demonstrated to contribute to the stimulation of angiogenesis. The hypoxic conditions within the developing tumour stimulate malignant cells to enhance the production of exosomes. Normal endothelial cells uptake hypoxia-induced exosomes, which subsequently encourage the development of new angiotubes. This process ultimately leads to the establishment of a network of new blood arteries (95).

For instance, the metastasis of breast cancer is a complex and gradual process in which cancer cells deviate from their normal activity and spread from the initial location. They enter the lymphatic system and bloodstream, and finally settle in distant areas (96). Mounting data indicates that exosomes have a crucial function in regulating the course of breast cancer and its aggressive characteristics. TDEs have demonstrated the ability to control the establishment of pre-metastatic environments, the tendency of cancer cells to spread to specific organs, the movement and penetration of cancer cells, the maintenance of stem cell properties, and the ability of cancer cells to survive (97). **Table 1** represented various functions of TDEs in cancer.

**Table 1:** Functions of tumour-derived exosomes in cancer.

Exosome component	Cancer	Targeted cells	Function	Ref
**miR-122**	Breast CA	Lung fibroblasts neurons	↑ metastasis	(98)
**EGFR vIII**	Glioblastoma	Glioblastoma cells	↑ tumour growth	(99)
**FasL**	HNC	Activated CD8+ T cells	Induces apoptosis	(100)
**IL-6, fibronectin**	Multiple myeloma (BM-MSC)	MM cells	↑ tumour growth	(101)
**CD39/CD73**	Various tumours	T cells, Treg tumour cells	↑ adenosine production	(102)
**MET**	Melanoma	BM progenitor cells	↑ tumour growth ↑ metastasis	(103)
**KIT**	GIST	Progenitor muscle cells	↑ Invasiveness	(104)
**Hsp 70**	Colon CA	MDSC	↑ Immune suppression	(105)

## 4 Exosomes in lung cancer

Over the years, the role of exosomes in lung cancer detection has gained popularity among researchers. This status quo came to be as exosomes mediate the traffic between the plasma membrane, where proteins, lipids, nucleic acids and metabolites diffuse in and out of the lung cells (106). It raised a question, while some of these exosomes played a part in triggering cancer cells, can the same mechanism be used to treat a cancer patient? Thus, by understanding the progression, metastasis, epithelial-to-mesenchymal transition (EMT) and the angiogenesis of the lung cancer, the role and relationship that exosomes have on lung cancer can be discussed.

### 4.1 Exosomes in lung cancer progression

**Figure 6:**
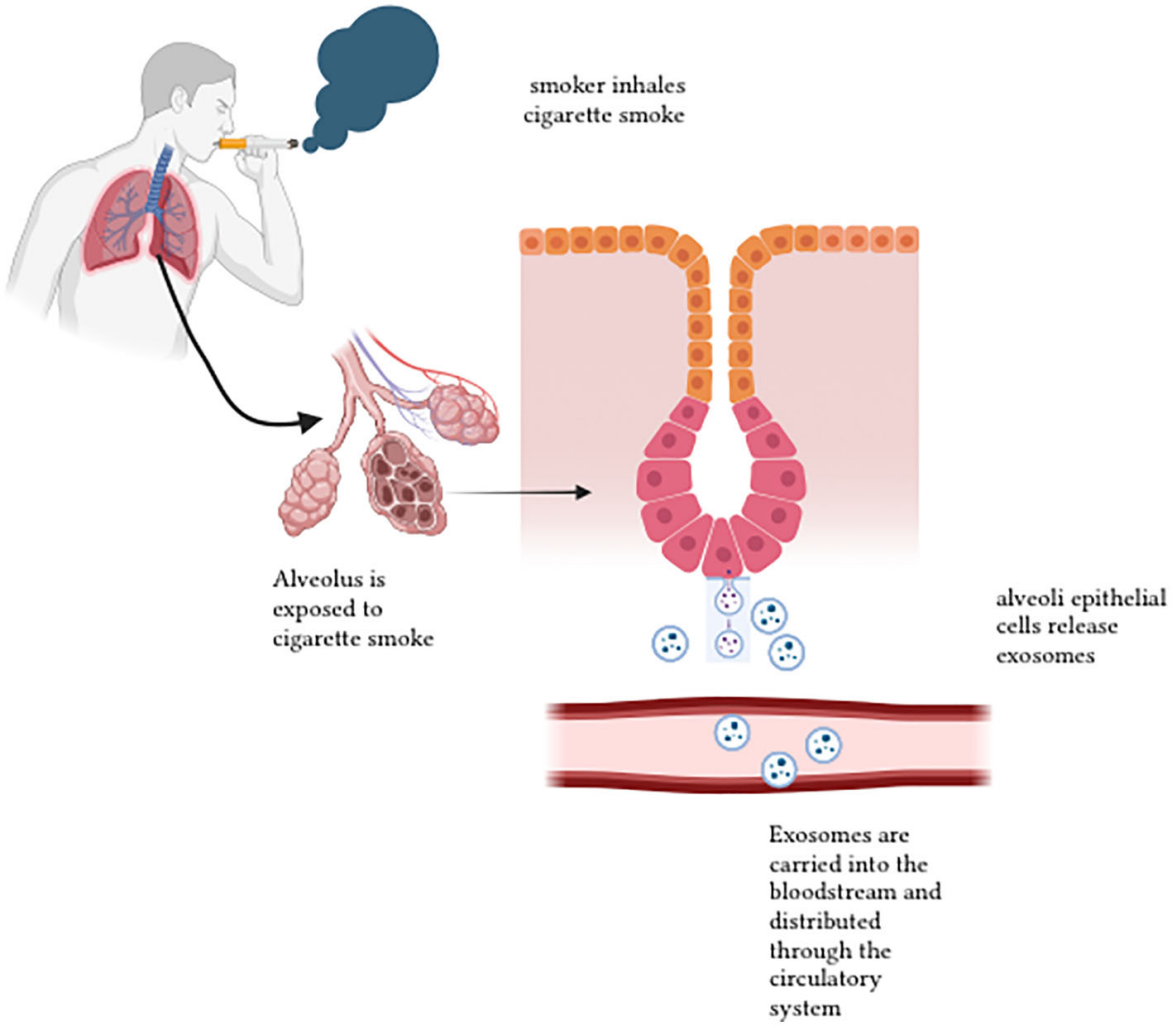
Release of exosomes after cigarette smoke exposure 1.

Most cases of lung cancer derived from exposure to cigarette smoke (107). A study done by Rizwan et al. describes the relationship of smoking and chronic diseases, which were mediated partially by EVs (82). Here, EVs are mentioned as exosomes and macrovesicles released by cells to the extracellular environment, where they carry cytosolic proteins, lipids and RNA. Zhong et al. emphasises that cigarette smoke induces EV release into the tissue cells and body circulation. The secretion of the EVs induces several biochemical and cellular processes, which include angiogenesis, endothelial dysfunction, tissue remodelling, pro-inflammation, oxidative stress, fibrosis and thrombosis (107). As a result, it triggers the pathogenesis of lung cancers related to cigarette smoke (CS). EVs contain proteins, nucleic acids and other molecules which reacts when there is a variation of the state of the parental cells (108). **Figure 6** demonstrates the release of exosomes after cigarette smoke exposure.

**Figure 7:**
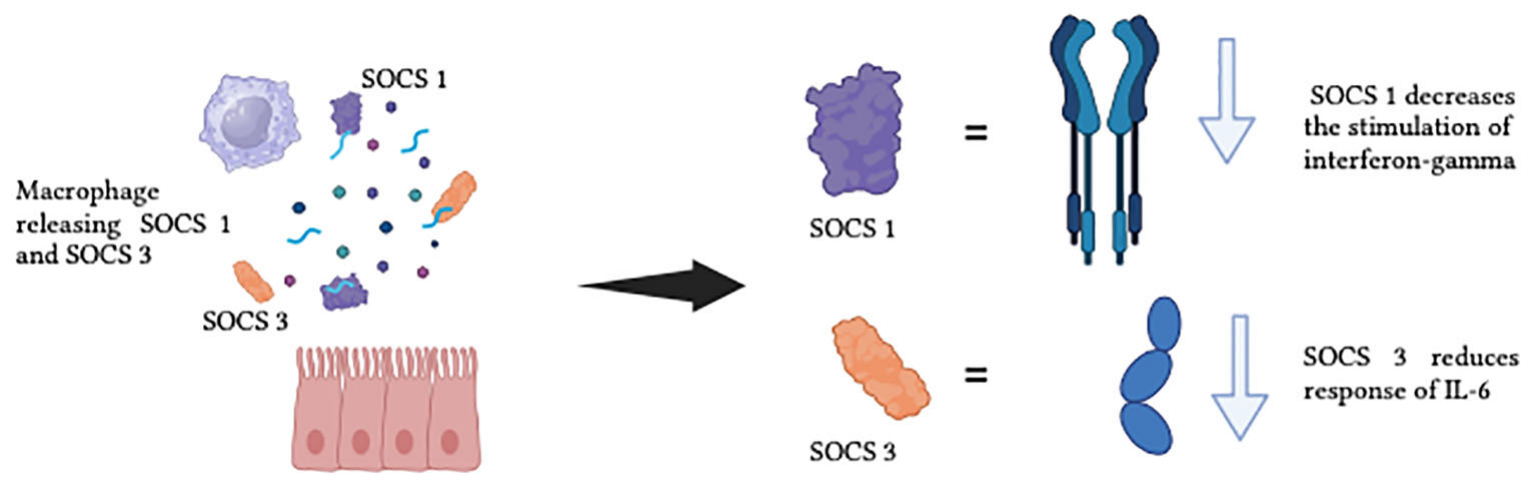
The function of SOCS1 and SOCS3 in normal state.

**Figure 8:**
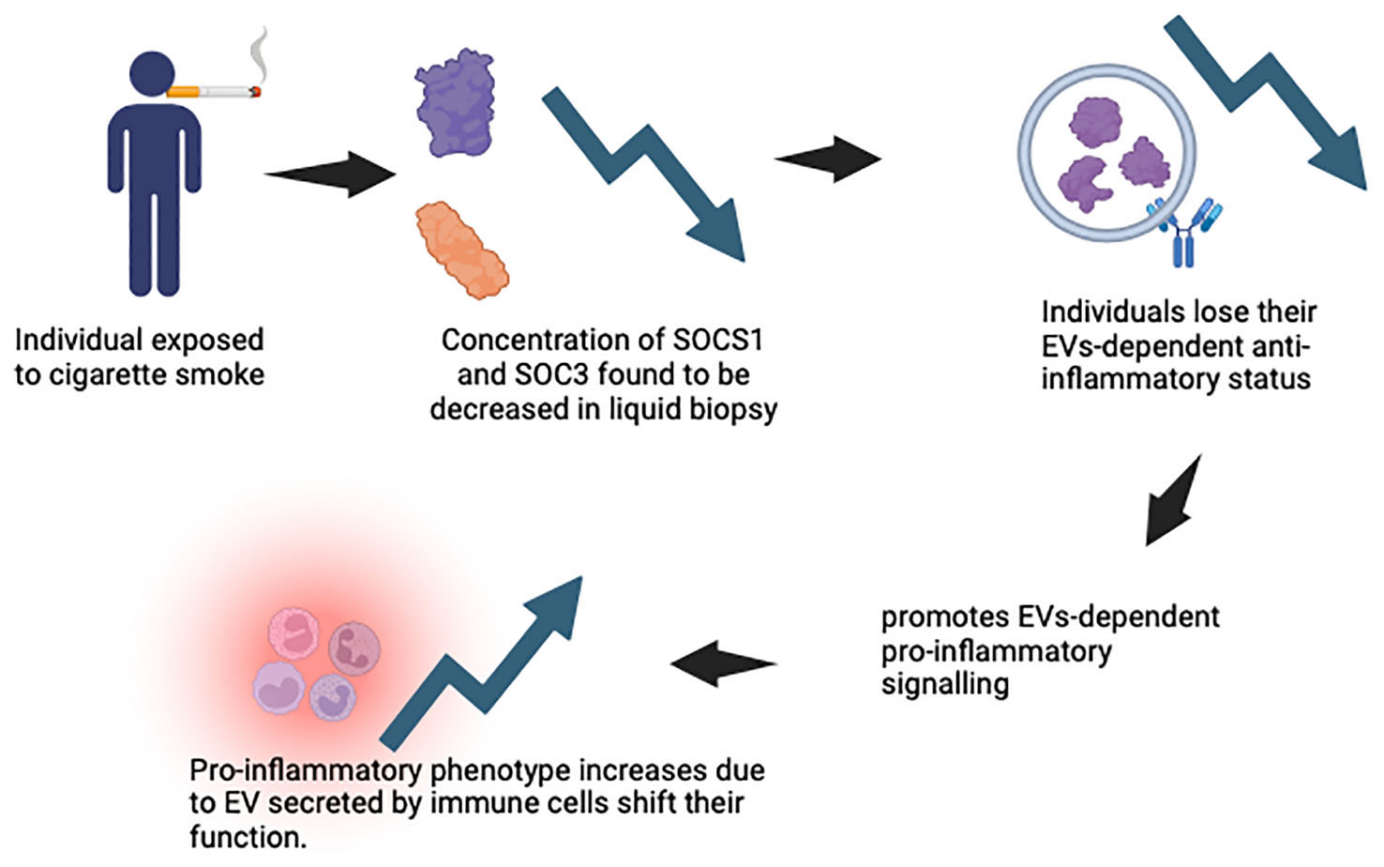
The concentration of SOCS1 and SOCS3 after cigarette smoke exposure.

The alveolar macrophages and the lung epithelium in healthy lungs act in a synergetic manner to expedite the removal of inhaled substances and allowing an anti-inflammatory state to be maintained. Cytokine signalling inhibitor protein 1 (SOCS 1) and cytokine signalling inhibitor protein 3 (SOCS 3) are delivered to the epithelial cells by the alveolar macrophages’ EV, thus maintaining the anti-inflammatory state (109). SOCS 1 reduces the stimulation of interferon γ signalling through the signal transducer and activator of transcription 1, and SOCS 3 reduces the response of the interleukin-6 (IL-6) signalling via the signal transducer and activator of transcription 3 (STAT 3). **Figure 7** demonstrates the function of SOCS1 and SOCS3 in normal state. When humans are exposed to CS, the concentration of SOCS in the liquid biopsy was reduced, which indicates that smokers lose their EVs-dependent anti-inflammatory status (109). CS also actively promotes EVs-dependent pro-inflammatory signalling associated with lung cancer, by shifting the function of EVs secreted by monocytes, epithelial cells, neutrophils, and endothelial cells from an anti-inflammatory to a pro-inflammatory phenotype. **Figure 8** illustrates the concentration of SOCS1 and SOCS3 after cigarette smoke exposure.

Alba et al. mentions that bioactive lipids such as sphingolipids (SLs), glycosphingolipids (GSLs), and epoxylane-like lipids play an important role in the aetiology of several diseases including cancer (110). Various studies found evidence of enhanced activity in lipid metabolism, especially in monounsaturated fatty acids and cholesterol (110). Generally, stem cells use energy derived from mitochondrial oxidative phosphorylation (OXPHOS) as the process generates more adenosine triphosphate (ATP) than glycolysis and produces the tricarboxylic acid cycle (TCA) intermediates utilised for macromolecule synthesis. The cancer stem cells (CSC) depend on specific signalling pathways to be regulated, and these pathways are influenced by environmental stresses such as inconsistent oxygen and nutrient levels, pH, inflammation and anticancer therapies (111). The CSC functions are regulated by a number of specific signalling pathways, which change in response to environmental stresses such as fluctuating oxygen and nutrient levels, pH, inflammation, and anticancer therapies. Cancers rely on angiogenesis, the fast proliferation of cancer cells, hypoxia and poor perfusion in tumours. Blood supply is often leaky and lacks a normal hierarchical structure, thus it poorly supplies nutrients and clearance of waste products to the cancer cells. The pathways involved includes cancer stem cell metabolism, aldehyde dehydrogenase metabolism, lipid metabolism and *de novo* lipogenesis. Begicevic et al. states that components related to the pathways of cancer cells are lipid droplets, monounsaturated fatty acids/stearoyl-CoA desaturase 1 (SCD1), 3-hydroxy-3-methyl-glutaryl-coenzyme A, Lipid biomolecules in CSCs (111).

The mechanism of EVs-mediated intercellular communication between human bronchial epithelial cells (HBECs) and lung fibroblasts (LFs) is based on the CS inducing relative upregulation of EVs miR-210 expression in HBECs, promoting the myofibroblast differentiation in LFs. As a result, the number of myofibroblasts is abnormally increased, thus promoting cancer development. The sensitivity of HBECs to cigarette smoke exposure (CSE), leads to the production of a large number of differentially expressed exosomal proteins (DEEPs), which are significantly high in cancer pathways, such as NF-κB p65, sulforaphane polysaccharide-1, and thioredoxin-interacting protein (111). This is evident of the strong effect of the development of DEEPs in tumours and metastasis. Nitrosamine 4-(methyl nitrosamine)-1-(3-pyridyl)-1-butanone (NNK) is an important component of CS. It has been reported by Xu et al. to be able to mimic epidermal growth factor, whereby it enhances cysteine protease activity via mitogen-activated protein kinase/extracellular regulated protein kinases (MAPK/ERK) signalling pathway. NNK was found to significantly possess enhanced μ- and m-calpain in NCI-H69 cell-derived EVs in a culture medium, which would have the capability to cleave the ECM, thus leading to increased lung cancer cell invasion and metastasis (112).

### 4.2 Metastasis and EMT of lung cancer

The regulation of CS in the expression of miRNAs in EVs is that the miRNAs can be internalised or transferred to neighbouring cells and elicit phenotypic effects similar to those of the parental cells. Exosomal miRNAs were found to affect cancer in two ways, which is the regulation of expression of protein-encoded oncogenes and tumour suppressors, such as anaplastic lymphoma kinase, tumour protein p53, vascular endothelial growth factor (VEGF) family, calcineurin-E; or as oncogenes and tumour suppressors such as let-7, miR-21, and miR-34 families (113). Exosomal miR-21 was proven to promote the malignant transformation of BEAS-2B. It also has a great potential to become a universal biomarker to diagnose cancer. A study found that in CSE-treated HBECs, the exosomal miR-21 levels were higher than those in untreated HBECs (113). When this happens, miR-21 in exosomes progresses STAT3 activation, thereby increases the VEGF levels in recipient cells. This process was implicated in the angiogenesis and malignant transformation of HBECs cancer patients.

In a study by Karacosta et al., it is found that in non-small-cell lung carcinoma (NSCLC), epithelial states became quite heterogeneous towards E-Cadherin, CD24, and MUC1 expression (114). Heterogeneity was also observed within the partial EMT states for various markers, and the expression of EMT-specific transcription factors during EMT was dispensable for certain cells. Phenotypically distinct transient cells were found featuring an alternative EMT program that involved protein internalization retained some epithelial identity and proliferative capacity (114). Let-7a, let-7b, let-7c, let-7f, let-7g, and let-7i were found to be significantly downregulated in CSE-induced HBEC-derived EVs (113). Héliot et al., exposed BEAS-2B to EVs from liquid cytology (BALF) isolated from 10 smokers and 10 non-smokers over 24 h. They found that let-7e, let-7g, and miR-26b were significantly reduced in EVs of smokers compared to non-smokers (115). The results were consistent with Chen’s experiment, where miR-21 and miR-27a expression were significantly increased in smokers’ EVs exposure to BEAS-2B and the expression of let-7e and let-7g was significantly decreased in the presence of smokers’ EVs. BEAS-2B exposure to smokers’ EVs was also found inducing the release of the pro-inflammatory cytokines IL-6 and IL-8, alongside the specific lung cancer biomarker cytokeratin 19 fragment antigen 21-1 (113). Moreover, TGF-β expression levels were found to be significantly increased in lung tumours than non-tumour control tissues, similar with the tumour expressions found in those of chronic obstructive lung disease (COPD) samples (116).

### 4.3 Angiogenesis in lung cancer

Exosomal miRNAs are involved in the development and tumour angiogenesis, which enhances the vasculature of the tumour. The specific exosomal miRNAs in question which regulate the angiogenic processes were found to be miR-296 and miR-132, which were induced by VEGF. A study found that exosomal miRNAs were upregulated by tumour cells in the endothelial cells, where miR-9 was found to promote the migration of endothelial cells and induce tumour angiogenesis as it was found that inhibition of miR-9 decreases tumour cell proliferation without causing apoptosis (117). Angiogenesis contributes to the significant development of lung cancer, where new blood vessels would be formed and distribute oxygen and nutrients to the quick growth of the tumour cells (117). As a result, tumour invasion and dissemination are developed. Tumour vascularisation initiates the angiogenic switch, where an ingrowth of blood vessels occurs in a tumour devoid of blood vessels. This process allows the malignant cells to gain access to the circulation system. Vascular homeostasis is regulated by a large number of pro-angiogenic and anti-angiogenic factors (118). Hyper-proliferation of tumour cells causes the increased oxygen consumption, and when the tumour mass exceeds the blood supply, the tumour turns hypoxic. Hypoxia gives rise to the production of pro-angiogenic factors, which leads to the enhanced, rapid and disordered blood vessel formation (118). Cellular adaptation to hypoxia is mainly temporised by a family of transcriptional regulators called the hypoxia-inducible factors (HIFs), which are heterodimers. They consisted of an oxygen-dependent α-subunit (HIF-α) and an oxygen-independent β-subunit (HIF-β). HIF-α has three isoforms, HIF-1α, HIF-2α, and HIF-3α. HIF-1α plays the major responsibility to activate transcriptional responses under hypoxia. Hypoxia-induced stabilization of HIF-1α, promotes the increase of several pro-angiogenic genes including VEGF, FGF and phosphogluconate dehydrogenase (PDG) (119). EV release was found to influence HIF1α and HIF2α activation, in which these increases following the onset of hypoxia (120). Vessel intussusception occurs, which is a development of intravascular growth mechanism, consisting of the splitting of pre-existing vessels into two new vascular structures (110). Angiogenesis also plays a role in the immunological response of the microenvironment. It results in the release of pro-inflammatory cytokines, which promote cell survival and proliferation. These cytokines will interact with immune cells, such as neutrophils and macrophages, enhancing the recruitment and infiltration of immune cells while activating signalling pathways that promote angiogenesis (118). This results in a violent cycle, where angiogenesis supports the inflammatory process, with the inflammatory process supporting angiogenesis. These immune cells can contribute to build new blood vessels through vasculogenesis. In addition, new research has emphasised the contribution of epigenetic alterations in lung cancer angiogenesis, specifically the influence of microRNAs towards pro- and anti-angiogenic factors (118). During a typical immune response, the system triggers self-tolerance that dissuades immune cells from attacking indiscriminately through immune checkpoints. Tumour cells will stimulate checkpoint targets to protect themselves from being attacked in cancer. Combining immune checkpoint inhibitors with angiogenesis inhibitors in a preclinical test showed promise in addressing the complex interaction between angiogenesis and immune responses in lung cancer, indicating the possibility of combination therapy to enhance patient outcomes (121–123).

## 5 Exosomal contents as diagnostic or prognostic biomarkers of lung cancer

Early detection and investigation of tumours is crucial for prompt and effective treatment. The most common method for detecting tumour-related biomarkers is liquid biopsy (124). Liquid biopsy utilises body-related fluids and is a less invasive method. The standard practice for detecting lung cancer in clinics is tissue collection. However, since this method provides a limited amount of tissue samples, has specific times of collection and has frequent tumour heterogeneity, it is not enough to present a complete picture of the disease (125). Thus, research has been conducted regarding alternative methods of diagnosing lung cancer. Accurate diagnostic and prognostic biomarkers are necessary to treat various cancers, including lung cancer. Exosomes are good candidates for use as lung cancer biomarkers. Because of their lipid bilayer encapsulation, exosomes are believed to offer a high degree of stability to the molecular cargo they carry, which makes them perfect for liquid biopsies (126). Lung cancer cell-derived exosomes (LLCDEs) carry possible biomarkers that can be used for the diagnosis of lung cancer. An extracellular array was created by Jakobsen et al. (2015) to measure the concentration of 37 proteins linked to lung cancer in exosomes (127). With just a 10-microliter plasma samples, the assay was found to be 75% accurate in differentiating between NSCLC patients and healthy people. The initial research on exosomes in lung cancer showed that pleural effusions of lung cancer patients contain exosomes that can be isolated for both diagnosis and prognosis (128). **Table 2** shows an exosomal components in lung cancer-cell derived exosomes.

**Table 2:** Exosomal components in lung cancer-cell derived exosomes.

Biomarker	Exosomal components	Clinical use	Clinical outcome	References
**miRNA**	miR-17-3p, miR-21, miR-106a, miR-146, miR-155, miR-199, miR-192, miR-203, miR-205, miR 210, miR-212, miR-214	Diagnosis;	Differentiate between NSCLC patients and healthy donors using qRT-PCR	(129)
	miR-378a, miR-379, miR-139-5p, miR-200-5p	Screening panel	Early detection through serum exosome analysis	(130)
	miR-151a-5p, miR-30a-3p, miR-200b-5p, miR-629, miR-100, miR-154-3p	Confirmation of cancer presence using multiplex assays	Improved diagnostic accuracy for various cancers	(131)
	miR-21, miR-4257	Predicting poor progression-free survival using exosomal miRNA levels	Identifies patients at higher risk of disease progression	(132)
	miR-21, miR-155	Recurrence prediction through miRNA profiling	Early identification of patients likely to experience recurrence	(133)
	miR-100-5p	Assessing cisplatin resistance via exosomal miRNA analysis	Helps in selecting appropriate treatment options	(134)
**Proteins**	EGFR	Detection of EGFR mutations in exosomes via liquid biopsy	Non-invasive diagnostic tool for EGFR mutation-positive cancers	(135) (136)
	NYESO-1, EGFR, PLAP	Evaluation of poor prognosis markers through exosome profiling	Indicates aggressive tumour behavior and poor patient outcomes	(137)
**RNA**	ALK-EML4	Identification of ALK-EML4 fusion genes in exosomal RNA	Early detection of ALK-positive NSCLC	(138)
	EGFR	Exosomal RNA-based EGFR mutation analysis	Enables early and non-invasive cancer diagnosis	(139)

miR, microRNA; EGFR, epidermal growth factor receptor; DNA, Deoxyribonucleic acid; RNA, Ribonucleic acid and ALK-EML4, echinoderm microtubule associated protein-like 4-anaplastic lymphoma kinase.

### 5.1 Diagnostic biomarkers

#### 5.1.1 Exosomal MiRNA as diagnostic biomarker

Several miRNAs from tumour-related exosomes are being assessed as biomarkers in the plasma of tumour patients in an effort to enhance the detection and management of cancer since irregular miRNA levels have been linked to a number of human malignancies like breast cancer, prostate cancer, ovarian cancer and many more. Moreover, TDE have been represented as the biomarkers of the miRNA expression from the originating tumour cells (140). Some researchers found circulating exosomal miRNAs that can be utilised for the diagnosis. It was stated that 12 of these exosomal miRNAs have been elevated in the bloodstreams of lung cancer patients in comparison to control samples (hsa-miR-17-3p/-21/-106a/-146/-155/-191/-192/-203/-205/-210/-212/-214) (141, 142). In another report, four exosome miRNAs (hsa-miR-378a,-379,-139-5p/-200b-5p) in plasma samples are assumed to be possible biomarkers for diagnosing lung adenocarcinoma (130). Compared to healthy patients, the exosome miRNA expression profile is very different in NSCLC patients, as miR-320d, -320c, and -320b are possible biomarkers to determine the effectiveness of immunotherapy in advanced NSCLCs (143). Moreover, early identification of NSCLC can also be done by tumour-derived exosome miRNAs (AD-specific miR-181-5p/miR-30a-3p/miR-30e-3p/miR-361-5p, and SCC-specific miR-10b-5p/miR-15b-5p/miR-320b) and serum exosome miRNAs (miR-146a-5p/miR-486-5p) which are very precise and non-invasive biomarkers (144).

Researchers examined miRNA panels of six circulating miRNAs in a study with the biggest number of participants to date (256 individuals suffering from lung adenocarcinoma), and they discovered that the sensitivity varied from 67% to 73% and specificity varied from 66% to 80%, correspondingly (145).

#### 5.1.2 Exo-GAS5 as diagnostic biomarker

Growth Arrest-Specific 5 (GAS5) is a prominent long non-coding RNA that plays a crucial role in regulating metabolism, cell death, and cellular development. GAS5 exerts its main antitumour effects by inhibiting cellular proliferation and promoting programmed cell death (apoptosis) in several types of malignancies. The phrase “exo-GAS5” refers to the long non-coding RNA GAS5 that is present within exosomes (146).

Reduced levels of noncoding RNA GAS5 in exosomes found in the bloodstream (Exo-GAS5) may serve as a non-invasive blood-based tumour marker for early-stage NSCLC diagnosis. The significance of GAS5 as a biomarker for the identification of early-stage NSCLC was investigated by Li et al., in 2019. Their findings showed a correlation between the levels of GAS5 in circulating exosomes and both tumour size and TNM stage (p < 0.05). Exo-GAS5 expression varied between NSCLC patients and controls (p < 0.001). Furthermore, their data demonstrated the diagnostic utility of Exo-GAS5. When it came to NSCLC diagnostic value, the Exo-GAS5 (AUC of 0.857) outperformed the CEA (AUC of 0.758) (147).

#### 5.1.3 MiR-17-5p combined with CEA, CYFRA21-1 and SCCA as diagnostic biomarker

The diagnostic and prognosis accuracy of lung cancer can be improved by incorporating miR-17-5p with conventional tumour markers including CEA (Carcinoembryonic Antigen), CYFRA21-1 (Cytokeratin 19 Fragment), and SCCA (Squamous Cell Carcinoma Antigen). It has the ability to increase the accuracy and precision of a test, improve its usefulness in diagnosing conditions, and offer valuable information on the effectiveness of treatments (148).

MiR-17-5p, which belongs to the miR-17-92 cluster, is recognised for its carcinogenic qualities. It is frequently increased in various types of cancers, including lung and breast cancer, and promotes cell growth while inhibiting cell death. CEA, a glycoprotein, is commonly used as a tumour marker for colorectal cancer, as well as in certain cases of breast and lung cancer, due to its involvement in cell adhesion (149). Cytokeratin19 is a protein found in epithelial cells and is broken down into fragments known as CYFRA21-1. It is commonly used as a tumour marker for NSCLC. SCCA is a protein associated with squamous cell carcinoma, which is found in epithelial cells. Squamous cell carcinomas utilise it as a biomarker (150).

The level of exosomal miR-17-5p when combined with CEA, CYFRA21-1and SCCA is a lot more in patients with NSCLC than in controls. In comparison to the healthy control groups, NSCLC patients exhibited far greater production levels of exosomal miR-17-5p, as reported in Zhang et al.’s research report. For exosomal miR-17-5p, the AUC value found by the authors was 74.6%. The AUC value rose to 84.4% when the miRNA was coupled with three established serological indicators for the identification of non-small cell lung cancer: CEA, CYFRA21-1, and SCCA (150).

#### 5.1.4 EGFR as diagnostic biomarker

Alterations in the epidermal growth factor receptor (EGFR) serve as crucial indicators for the detection and management of lung cancer, particularly NSCLC. EGFR is a transmembrane receptor tyrosine kinase that regulates cellular proliferation, survival, and differentiation. Alterations in the EGFR gene can lead to uncontrolled cell growth and the development of cancer. The level of EGFR expressed in the plasma of patients with lung cancer is significantly elevated compared to that of healthy individuals (151).

Exosomal RNA and DNA can be used identify any lung tumour related EGFR mutations (128). Recent studies used exosomal DNA and RNA for genetic testing of advanced lung adenocarcinoma a TP53, EGFR, PKD1, and ALK genes were successfully tested, which are the top 4 mutated genes mutated in this condition (128). The most common EGFR mutations that occur in NSCLS include Exon deletions and L858R point mutations. The most prevalent kind of EGFR mutation is exon 19 deletions, which make up approximately forty-five percent of all EGFR mutations in NSCLC (152). L858R point mutation is a substitution in exon 21 that is found in 40–45% of EGFR-mutant NSCLC cases (153). Mutations such as T790M, S768I, L861Q, and G719X have a lower occurrence rate but are of great therapeutic importance. These mutations are specifically related with resistance to first-generation EGFR inhibitors (154).

#### 5.1.5 Exosomes in malignant pleural effusions as diagnostic biomarkers

Malignant pleural effusion (MPE) is a frequent complication in patients with advanced lung cancer. It develops when cancer cells penetrate the pleural space and accumulate fluid. This fluid comprises not just cancer cells, but also several other components, such as exosomes and can be used as a diagnostic biomarker (155).

A study done to examine the exosomal miRNA of pleural effusion in lung adenocarcinoma and tuberculosis revealed that exosomal miRNA profiles in MPE may help in the differential diagnosis. They identified 99 upregulated and 91 downregulated miRNAs in malignant pleural effusion (156). Another research states that MPE’s high concentration of tumour-derived exosomes offers a chance for rapidly increasing the quantity of exosomes accessible for possible therapeutic and diagnostic uses (157). Elevated levels of exosomal miR-21 and miR-30a in MPE are associated with lung cancer and could aid diagnosis as well (158).

### 5.2 Prognostic biomarkers

Exosomal components can be used as non-invasive prognostic biomarkers of lung cancer. Exosomal membrane bound proteins like NY-ESO-1, PLAP, EGFR, Alix and EpCam are connected to the overall survival (OS) of NSCLC patients suggesting that these proteins can be prognostic biomarkers along with exosomal miRNA (159).

#### 5.2.1 Exosomal MiRNA as prognostic biomarker

Findings of exosomal miRNA tests in the bloodstreams of patients with prostate, liver, and myeloma cancers have demonstrated clinical significance in predicting the patients’ prognosis. likewise, exosomal miRNA profiles can offer trustworthy information about lung cancer detection and treatment. Poor OS of NSCLC has elevated exosomal miR-10b-5p, miR-23b-3p and miR-21-5p associated with it. Serum exosome miR-146a-5p inhibition reflects the impact of cisplatin on NSCLC and denotes a poor progression free survival (PFS) (160) Throughout therapy, the T-cell suppressor miR-125b-5p found in NSCLC plasma exosomes is decreased, suggesting that patients’ T-cell function has enhanced and they would respond well to immunotherapy (161).

The downregulation of miR-29a-3p and miR-150-5p in exosomes that are released are biomarkers that is correlated with the amount of radiation that is administered and may eventually be employed to foresee unanticipated side effects, like toxicity (162). Recurring cancers like colorectal cancer and breast cancer can have a considerable upregulation of miR-21 and miR-155 in comparison to the initial tumours. These two miRNAs are also elevated in the serum exosomes of animals with tumours that reoccur. In patients with NSCLC, the increased levels of miR-21 and miR-4257 in plasma exosomes may serve as a prognostic biomarker for recurrence. All of these earlier are very positive and offer a new, less intrusive approach for the prognosis of lung cancer (163).

#### 5.2.2 Exosomal protein as prognostic biomarker

Numerous types of exosomal membrane-bound proteins have been discovered and recognised as possible prognostic biomarkers for lung cancer. Several noteworthy examples include NY-ESO, PLAP, EGFR, Alix and EPCam. NY-ESO is a very prevalent cancer antigen found in tumours but not in healthy tissues, making it a more precise indicator for cancer prognosis. The existence of this exosome has been associated with unfavourable prognosis in patients with NSCLC. Elevated levels of placental Alkaline Phosphatase (PLAP) suggest advanced stages of cancer and are associated with poor overall survival (OS) in patients with NSCLC. Exosomal EGFR can be used to assess the progression of the disease and anticipate the patient’s reaction to the required treatments (151). Alix is a protein that plays a role in the process of exosome formation. The presence of Alix in exosomes is indicative of the tumour’s aggressiveness and is associated with unfavourable clinical outcomes. EpCam (Epithelial Cell Adhesion Molecule) is linked to the metastasis and lower survival rates in people with NSCLC (164).

### 5.3 Challenges in clinical translation of exosomal biomarkers for lung cancer detection

The pre-clinical stage of biomarker exploration for LCCDEs is still ongoing. They remain a barrier to the clinical translation of these studies, despite the encouraging results for their use in screening initiatives. Using circulating exosomal miRNA profiles in blood requires caution, even if their value as lung cancer diagnostic and prognostic indicators is always expanding. Even while exosomal RNAs are extremely helpful, it can be difficult to find them in early-stage malignancies because of their low expression levels. Furthermore, there is currently no officially established tool for isolating exosomes from clinical samples. Lastly, in order to demonstrate their clinical usefulness, sizable prospective clinical trials are required. However, if all of these issues are fixed, LCCDEs could soon be considered as potential lung cancer indicators (140).

## 6 Therapeutic potential of exosome

In this section, we discuss three potential therapeutic applications of exosomes in lung cancer: (1) exosome-mediated drug delivery system; (2) exosome-based immunotherapy; (3) exosomes as therapeutic targets.

### 6.1 Exosome-mediated drug delivery system

Exosomes are naturally occurring vesicles that are released by different bodily cells and play a vital role in the communication of information between cells. Exosomes are broadly distributed with low-toxic carriers of genetic material *in vivo*. Additionally, compared to other delivery methods like liposomes or nanoparticles, they have a cellular absorption that is more than thirty times higher (165). Exosomes are also capable of withstanding extreme environmental factors, like a low blood pH, which makes them a viable biocarrier for drugs, nucleic acids, and imaging agents in cancer treatment. There is a lot of interest in the potential of exosomes as anticancer drug delivery vehicles, especially for agents with low solubility and limited off-target delivery.

Exosome manipulation, such as blocking their synthesis or altering their surface membranes, has shown great promise in slowing the spread of tumours and their metastasis when loaded with cargo like proteins, nucleic acids, or medications. Notably, as of 2022 (https://www.clinicaltrials.gov/), there were 118 active clinical trials involving exosomes for a variety of medicinal uses. Even though preclinical research has demonstrated encouraging therapeutic effects, ongoing clinical trials are still assessing these medicines’ potential. A detailed summary of clinical trials that use exosomes as therapeutic agents is given in **Table 3**. Though EVs have great potential for both therapeutic and diagnostic applications, there are still a number of important issues that need to be resolved.

**Table 3:** Overview of exosome-based clinical trials being conducted to treat cancer.

Exosome source	Disease	Type and Purpose of Intervention	Clinical trial identifier and phase	Sponsor	Status/Date completed or estimation of completion
Ultrasound-guided portal venous blood exosome	Pancreatic cancer	• Cohort; Observational study • To explore the feasibility and safety of sampling portal venous blood, analysing mRNA markers	NCT03821909	Ying Lv	Completed (2020) (166)
Curcumin conjugated with plant exosomes	Colon cancer	• Interventional study • Comparing exosome-loaded curcumin on immune modulation, phospholipid profile of normal and malignant patients	NCT01294072 (Phase 1)	University of Louisville	Recruiting (Estimation on completion 2024)
Blood samples	Sarcoma	• Cohort; Observational study • Evaluation of cancer pathogenesis, progression, and treatment efficacy of exosomes	NCT03800121	Centre Georges Francois Leclerc	Recruiting (Estimation on completion 2025) (167)
Urine exosomes	Prostate cancer	• Cohort; Observational study • Validation of non-digital rectal examination (DRE) exosome gene expression test of prostate cancer in biopsy	NCT02702856	Exosome Diagnostics, Inc.	Completed (2015) (168)
Blood samples from patients	Pancreatic cancer	• Cohort; Observational study • To isolate and analyse exosome, and to evaluate whether exosome activity has a connection to disease occurrence and outcomes in patients	NCT02393703	Memorial Sloan Kettering Cancer Center	Recruiting (Estimation on completion 2025) (169)
Blood samples	Lung metastasis osteosarcoma	• Cohort; Observational study • Identification of levels of circulating exosomal RNA with or without lung metastasis	NCT03108677	Ruijin Hospital, China	Active, Not recruiting (last update in clinical trial: 2023) (170)
Exosomal blood samples	Gallbladder carcinoma	• Cohort; Observational study • Establishing a correlation between exosome biomarkers and gallbladder carcinoma	NCT03581435	liu yingbin, China	Unknown status (last update in clinical trial: 2018)
Mesenchymal stromal cells-derived exosomes with KRAS G12D siRNA	Stage IV pancreatic adenocarcinoma	• Interventional study • Mesenchymal-derived exosomes with KRAS G12D in treating individuals with pancreatic cancer with KRAS G12D mutation	NCT03608631 (Phase 1)	M.D. Anderson Cancer Center, US	Active, not recruiting (Estimation on completion 2025) (171)
Portal vein blood	Pancreatic ductal adenocarcinoma	• Cohort; Observational • Test 3 CTC isolation methods and analyses for onco-exosomes in pancreatic cell culture media by flow cytometry	NCT03032913	University Hospital, Bordeaux, France	Completed (2017) (170)
Oral mucositis, head and neck cancer	Grape extract exosomes	• Interventional • To evaluate the ability of plant exosomes to prevent oral mucositis in head and neck cancer	NCT01668849 (Phase 1)	University of Louisville, US	Completed (2022)
Non-small cell lung cancer (NSCLC)	Plasma exosomes	• Interventional • To explore the consistency analysis of PD-L1 expression level detected in cancer tissues and plant exosomes	NCT02890849	Xinqiao Hospital of Chongqing, China	Completed (2019) (172)
Non-small cell lung cancer (NSCLC)	Dendritic-derived exosomes	• Interventional • Researcher propose a maintenance immunotherapy in 47 advanced unresectable NSCLC patients responding or stabilised after induction chemotherapy with Dex-based treatment to improve PFS rate at 4 months in these patients	NCT01159288 (Phase 2)	Gustave Roussy, Cancer Campus, Grand Paris, France	Completed (2015)
Thyroid cancer	Urine exosomal thyroglobulin and galectin3	• Cohort; Observational • Identifying urinary exosomal proteins (thyroglobulin and galectin 3)	NCT03488134	National Taiwan University Hospital, Taiwan	Completed (2024) (173)
Colon cancer	Blood samples	• Cohort; Observational • To explore novel ways of diagnosing and predicting the spread to other organs, such as live	NCT03432806	Memorial Sloan Kettering Cancer Center, US	Active, not recruiting (Estimation on completion 2025)
Urine samples	Prostate cancer	• Interventional • To investigate the utility of a validated urine test which predicts the likelihood of high-grade prostate cancer on an initial prostate biopsy	NCT03235687	Exosome Diagnostics, Inc., US	Unknown status (Last update on 2022) (169)
Serum exosomes	Triple-negative breast cancer	• Interventional • Assessing response to pembrolizumab in the primary tumour, circulating lymphocytes	NCT02977468 (Phase 1)	Eileen Connolly, US	Recruiting (Estimation on completion 2025)
Urine exosomes	Thyroid cancer	• Cohort, Observational • Evaluation of new therapeutic mechanisms and medications for poorly differentiated or anaplastic thyroid cancer	NCT02862470	National Taiwan University Hospital, Taiwan	Completed (2020)
Blood samples	Lung cancer	• Cohort; Observational • To promote the rational use of liquid biopsy in the clinical detection of lung cancer through drug efficacy, surgical effect evaluation, recurrence monitoring, prognosis judgment, and molecular differentiation by analysing blood ctDNA	NCT03317080	West China Hospital, China	Active, not recruiting (Estimation on completed 2023) (174)

#### 6.1.1 Advancements in bioengineering technology

**Figure 9:**
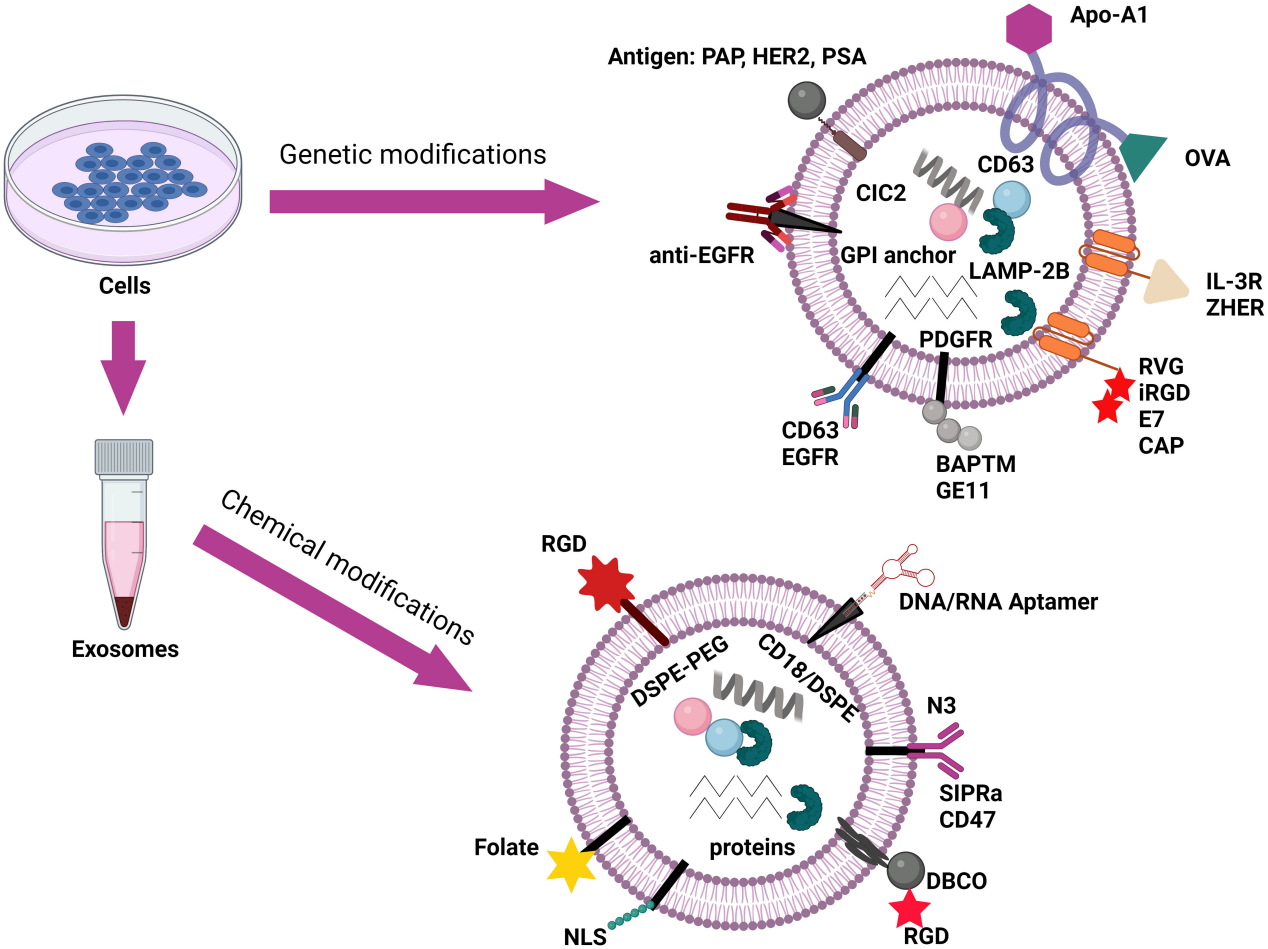
Schematic representation of exosome surface functionalization via genetic and chemical modifications for targeted therapeutic delivery. Adaptation from Akbari et al. (2023) (176).

Exosomes encapsulate therapeutic agents through common methods such as direct loading (electroporation, extrusion, and sonification) and indirect loading (modification of donor cells to produce specific exosomes) (175). However, natural exosomes lack the target selectivity and distribution mechanism required to achieve meaningful impacts. Several advancements have been studied to examine its impact on functionality and effectiveness of exosomes in targeting cancer cells. One method is to modify the surface of exosomes by attaching ligands or other antibodies to allow them to bind to specific receptors. This can be done either through genetic or chemical modification (**Figure 9**). Vandergriff et al. (2018) bonded exosomes with cardiac homing peptides (CHP) through a streptavidin-biotin linkage, giving it the ability to bind to infarcted heart tissue. This was evaluated on rat models with ischemia/reperfusion injury, and higher levels of exosomes were found in the cardiac sections of neonatal rat cardiomyocytes (177). Grzesik et al. (2023) and Ferber et al. (2017) improved on the natural removal of exosomes from the circulation by inhibiting glycan-binding proteins. The former used a metabolic glycoengineering approach, resulting in exosomes that bind to dendritic cells and evade immune clearance, whereas the latter coated exosomes with linear polyglycerol sulphate to allow for specific targeting of mannose moieties while avoiding immune-targeting glycans (178). These studies show how the properties of the exosomes themselves are altered through genetic modifications. Alternatively, chemical modification focuses on direct conjugation of molecules to the exosomal surface (**Figure 9**) (176). According to Li et al. (2023), exosomal integrins such as α6β4 and α6β1 have shown to target lung epithelial cells facilitating metastasis. This suggests that altering exosomal integrins changes their biodistribution profile providing further options in improving the lack of target specificity of exosomes (179).

Microfluidic technology has demonstrated a pivotal role in the manipulation of drug delivery of exosomes. This enables the conjugation of a variety of ligands to exosomes, including aptamers, graphene oxide/polydopamine (GO/PDA), PEG-ylated lipids, and biointerfaces. They provide greater stability and biocompatibility to exosomes and allow researchers to trace the physical properties of cancer cells through target-specific cancer populations (180). Development in technologies such as the atomic force microscopy (AFM), and localised surface plasmon resonance (LSPR) allows for better visualisation and characterisation of these tumour-derived exosomes (175). For example, Ballard et al. (2020) used Fe-65 engineered exosomes to deliver Corynoxine B to the brain to treat Alzheimer’s disease (AD) using animal models which showed promising results and might pave the way to an alternative treatment to AD (181).

#### 6.1.2 Advancements in nanotechnology

Nanoparticles are possible carriers that have demonstrated great promise due to their small size, huge surface area, and ability to attach targeted molecules. The integration of nanoparticles into exosomes allows them to deliver therapeutic substances to specific cells or organs (175). Nanoparticles target cancer tissues through the use of passive and active targeting along with a phenomenon known as enhanced permeability and retention (EPR effect) (182).

Gold nanoparticles (GNPs), in particular, show potential for cancer cell delivery. GNPs are synthesised in the lab along with biopolymers like polyethylene glycol (PEG). This PEG later forms an inert hydrophilic surface, which prevents aggregation. This addition enhances its hydrophilicity by enhancing its stability in biological settings and at high salt concentrations. Furthermore, it prevents recognition by the mononuclear phagocyte system (MPS), which would otherwise remove AuNPs from circulation, hence increasing their half-life in the bloodstream and biodistribution (183). Previous study demonstrated conjugated GNPs with doxorubicin (an anticancer drug) which showed cytotoxicity against cancer cells through cell viability analysis (14). Betzer et al. (2017), conducted a study using mouse models with brain disorders and GNPs for labelling. They used neuroimaging to track the exosomes and delivered the GNPs through an active, energy-dependent mechanism mediated by GLUT-1. Increased accumulation of GNPs was observed in the lesion site indicating its diagnostic capabilities (184).

Advancements in nanoparticles have shown great promise in exosome-mediated drug delivery systems, but an important objective is to prioritise safety through minimising the amount of nanoparticles used. For these reasons, the use of nanoparticles in drug delivery systems necessitates careful control over their size, as well as stability under high protein and salt concentrations. They should not be smaller than 10 nm in order to maintain optical and electrical properties and avoid kidney excretion. To avoid the immune system and nonspecific interactions with other molecules, its charge must also be neutral or negative (183).

#### 6.1.3 Exosomes as tumour vaccines

Exosomes have potential use in cancer immunotherapy as tumour vaccines due to their crucial role in cancer immunotherapy. These vesicles can carry tumour-associated antigens (TAAs) and present them to T cells, thereby directly stimulating the immune system to target and eliminate cancer cells. This mechanism has paved the way in the development of exosome-based vaccines, specifically dendritic cell-derived exosomes (DEXs). DEX offer advantageous features such as extended stability and ease of engineering. Furthermore, they can express various proteins such as cytokines, receptor for cytokines, integrins, lectins and ligands for T- and B-cells. There are also tumour-derived exosomes (TEXs) which carries a wide range of TAAs which allows them to influence the behaviour of the immune cells through modulating gene expression to either enhance or suppress immune response.

Apart from their ability to affect the immune response, exosomes can also be used as drug carriers to deliver immune-boosting agents or therapies directly to tumour sites. This would be beneficial in not only improving the efficiency on the elimination of cancer cells, but also in sustaining tumour suppression. The clinical potential of exosome-based tumour vaccines is still being explored, but promising results have shown their capabilities in long-term immune memory, reducing immune evasion by tumours, and improving patient outcomes in various cancers (185).

### 6.2 Exosome-based immunotherapy

Tumour-related exosomes can be categorised into tumour-derived exosomes (TDEs) and tumour-associated exosomes (TAEs). The former refers to exosomes that are specifically secreted by the tumour cells while the latter can be secreted by tumour cells as well as cell types associated with the tumour cell (such as stromal cells, endothelial cells, immune cells) (186). Both of them can carry certain molecules that can induce tumour growth and metastasis in lung cancer.

#### 6.2.1 Epithelial-mesenchymal transition

TME is filled with cancer cells that could all secrete exosomes that affect the immune system to promote lung cancer cells. Epithelial-mesenchymal transition (EMT) is a biological process that results in the production of mesenchymal (fibroblast-like) cells with changes in their loss of cell-cell adhesion and polarity properties (187). This production is caused by exosomes, containing ZEB1 mRNA and SNAI1 binding to epithelial cells which are secreted by lung cancer mesenchymal cells and cancer-associated fibroblasts respectively. The mRNA promotes the transformation from epithelial cells to mesenchymal phenotypes and creates resistance to apoptosis, and promotes tumour growth (188).

#### 6.2.2 Effector T cells

**Figure 10:**
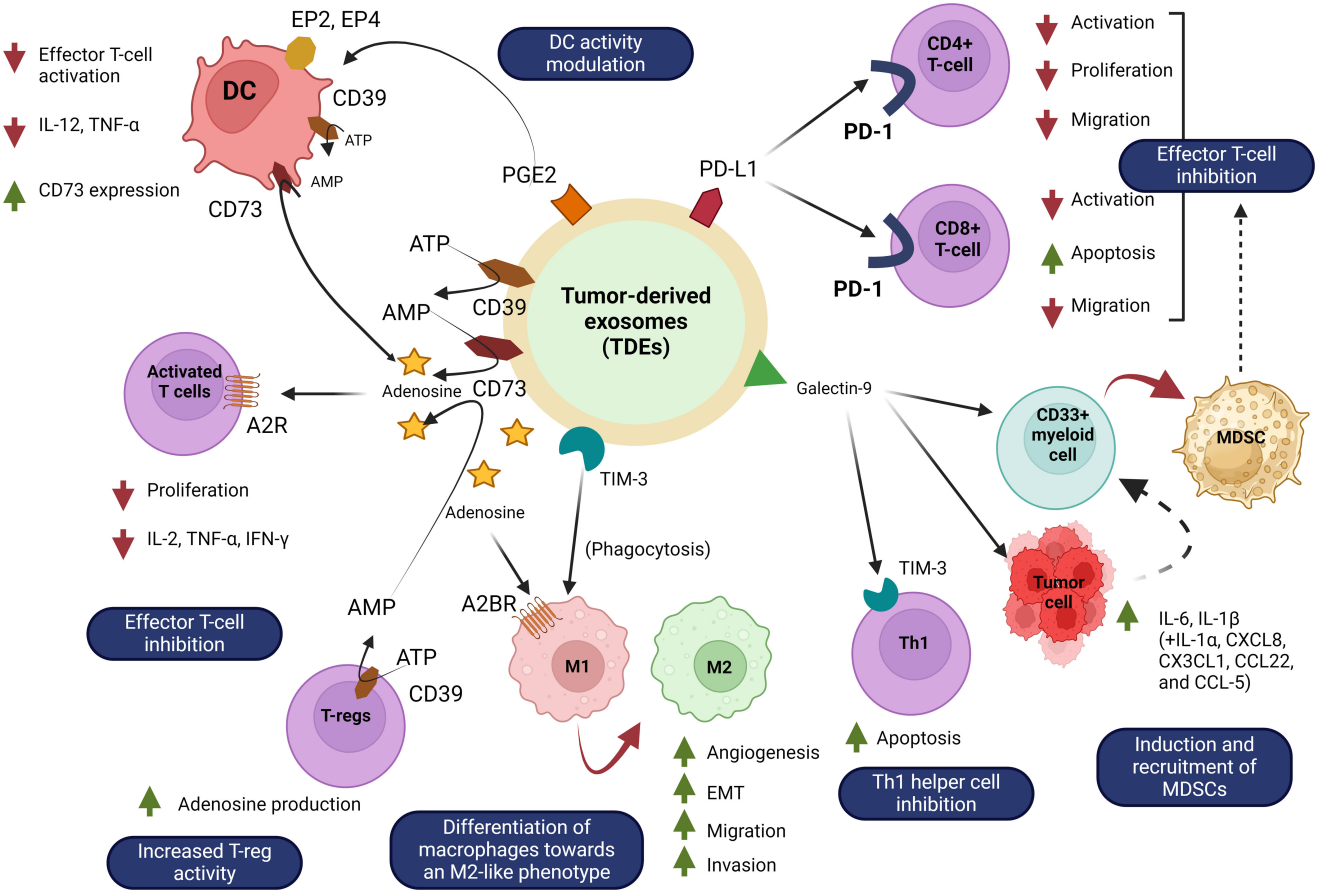
Mechanisms of immune suppression by tumour-derived exosomes (TDXs) in the tumour microenvironment. Adenosine can be produced from extracellular ATP by TDE-bound CD73/CD39. In the TME, adenosine is a molecule with immunosuppressive characteristics. Adenosine can directly prevent T-cell activation by attaching to its adenosine receptors (A2R, A2BR) on T-cells. Additionally, adenosine encourages macrophage differentiation toward an M2-like phenotype. By activating PGE2 receptors (EP2, EP4), TDE containing prostaglandin E2 (PGE2) can also increase dendritic cell (DC) production of CD73 and consequently adenosine. More adenosine is produced as a result of this. Furthermore, TIM-3 coupled to TDE can encourage macrophages to adopt a pro-tumoural M2-like phenotype upon phagocytosis. Galectin-9 linked to TDE can attach to TIM-3 on Th1 cells, causing the cells to undergo apoptosis. By stimulating the release of IL-6, IL-1β, and other cytokines by nasopharyngeal carcinoma cells and myeloid cells, TDE-bound galectin-9 can also encourage the transformation of myeloid cells into tumour-favorable MDSCs. Furthermore, effector T-cell migration, proliferation, and activation, including CD4+ and CD8+, can be inhibited by TDE-bound PD-L1.Adaptation and modification from Vautrot et al. (2021) (189).

Lymphocytes, natural killer (NK) cells, T cells, and B cells are all critical components of the immune system, particularly effector T cells such as CD4+ and CD8+. They have a vital role in the antitumour system, and TDEs are capable of modulating their activity by blocking their signalling molecules, which promotes apoptosis (for CD8+ T cells) while reducing proliferation, migration, and activation (**Figure 10**). Exosome-derived RNA has also been found in studies to bind to normal CD8+ T lymphocytes, causing phenotypic modifications that affect or limit their activity (190). Certain exosomes affect the Fas/FasL signalling pathway via MHC (major histocompatibility complex) class I molecules, which is a vital regulator of T cell death. In **Figure 10**, exosomal programmed death-ligand 1 (PD-L1) can interact with PD-L1 receptors on CD8+ T cells, leading to reduced secretion of cytokines, such as tumour necrosis factor-alpha (TNF-α), and interleukin-2 (IL-2), interferon-gamma (IFN-γ) (89, 190). These cytokines are important in the cell-to-cell communication, immune system regulation, and inflammatory of the body and a significant reduction of them would allow lung cancer cells to progress and metastasise.

#### 6.2.3 MDSCs and macrophages

As illustrated in **Figure 10**, TDEs and TAEs cause macrophages to differentiate into tumour-associated macrophages (M2). This is mainly due to proteins such as Hsp72 and RNAs from exosomes binding through pattern recognition receptors (PRRs), resulting in the secretion of proinflammatory cytokines such as TNF-α and interleukin 6 (IL-6) (191). Myeloid-derived suppressor cells (MDSC) is an immune suppression factor that includes immature myeloid cells (IMCs) that can be found in the TME (refer to **Figure 10**). Under normal conditions, IMCs are supposed to differentiate into granulocytes, dendritic cells (DC), or monocytes in the bone marrow. However, under a pathological environment, the maturation and differentiation stage of the IMCs are blocked. This results in an abundance of suppressive molecules to be secreted such as nitric oxide synthase (iNOS) and reactive oxygen species (ROS). These molecules directly inhibit the effector T cells, facilitate M2 macrophages, expansion of Tregs, and differentiation in Th17, facilitating lung cancer growth. There are several possible biomolecules that can promote MDSCs in lung cancer, mainly miR-21a containing exosomes are responsible, but others such as miR-9 and miR-181a could affect it as well (89).

#### 6.2.4 Natural killer cells and dendritic cells

NK cells are the initial step of the protective immune system, capable of directly eliminating cancer cells. Tumour-related exosomes disrupt the function and number of NK cells via many routes. One of the most significant is NKG2D, a receptor that binds to MHC class I and stimulates T cells, resulting in an antitumour immune response. In mesothelioma, cancer cells release exosomes carrying NKG2D ligands, which block its expression, resulting in decreased cytolytic activity in NK cells. Studies have shown that in mice models that were treated with TDEs had a low percentage of NK cells in their lungs (191). Dendritic cells are responsible for the initiation of T cells in response to cancer cells. Studies have demonstrated the immunosuppressive effect TDEs and TAEs have on these cells through inhibiting the differentiation of its precursors through prostaglandin E2 (PEG2) and cyclooxygenase-1 and 2 (COX-1 and COX-2), resulting in reduced DCs in the TME (89). In addition, these exosomes can be engulfed by dendritic cells causing it to become specialised into tolerogenic DCs that would release inducted tumour antigen specific regulatory T cells (Tregs) which can affect effector T cells, as well as inhibit the proliferation of Th1 cells (192).

### 6.3 Exosomes as therapeutic targets

#### 6.3.1 Removal of exosomes from the peripheral circulation

Due to the importance of exosomes in terms of tumour growth and immunosuppression, the removal of exosomes is an approach worth considering. This can be done through a process called extracorporeal hemofiltration through a machine called Aethlon ADAPT (adaptive dialysis-like affinity platform technology). This equipment works similarly to kidney dialysis where plasma and blood cells would eb passing through dialysis cartridges which are hollow (<200 nm wide) lined with immobilised affinity matrix. In order to filter out exosomes, the affinity agents used would be through aptamers, monoclonal antibodies, or various ligands depending on the specific exosomes. Several studies have used this technique and the removal of exosomes have been successful where lectins, HER2 positive exosomes have been filtered (193). However, there are concerns with the immunity related adverse side effects that can occur due to the importance of exosomes in normal physiological conditions. Exosomes are used within the body for cell-to-cell communication even among healthy cells, therefore further testing would be required in order to ensure the safety of using this technique (194).

#### 6.3.2 Inhibition of exosome production and secretion from cancer cells

Another approach is to inhibit either the production or secretion of exosomes from cancer cells. This can be achieved through genetic modification of said exosomes through short interfering RNA (siRNA). In this approach, the siRNA would bind to tumour cells coupled with the PD-L1 antibody on its surface, thereby inhibiting them and promoting apoptosis of those cells. Man-made exosomes would need to be used to deliver these siRNA to the target cells (195). A study conducted by Zhao et al. (2019), attempted this approach using biomimetic nanoparticles (CBSA) conjugated with siS1000A4. They demonstrated that the target specificity of the exosome allowed for a high affinity for the cancer cells, hence it was able to cause a significant gene silencing effect, resulting in the inhibition of the malignant cancer cells (196). This shows that inhibition of the exosomes is a more promising approach. Although, there are some challenges to this approach in terms of clinical translation. The availability of biofluids for making these exosomes varies due to the amount of exosomes being inconsistent in each sample. In addition, exosome preparation should be of homogenous, high quality, and contamination-free from other cellular vesicles (128). Therefore, exosomes currently require challenging hurdles to produce on a large scale. There are other options such as proton pump inhibitors, a common medicine to relieve gastric acid. Due to its principle of inhibiting vacuolar H^+^ ATPases, it can suppress the release of pacific and vesicle-like structures by tumour cells. There are other drugs such as dimethyl amiloride (DMA), ceramide, and omeprazole which have demonstrated suppression of exosome secretion when targeted (193). However, this also brings in the challenges since they require exosomes to be specifically delivered to their target cells. Exosomes are cellular structure that shows great potential in being developed into a possible treatment option for lung cancer, but further testing needs to be conducted in order to validate their safety and improve on loading and separation methods that would allow for them to be mass produced.

## 7 Conclusion and future perspective

The breakthrough in exosome research during the past two decades has been attributed to the rapid advancement of biological tools. Exosomes play a significant role in lung cancer immunological modulation, treatment resistance, and tumour metastasis as a component of TME. Understanding the impact of exosomes derived from lung cancer on the extracellular milieu would offer fresh perspectives on lung cancer treatment. New approaches are appealing and have the potential to change the landscape of lung cancer treatment. Examples of these approaches include the removal of particular exosomes to impede the growth of tumours and the transfection of exosomes with particular miRNAs to reverse drug resistance. Exosomes offer special benefits as therapeutic delivery systems and biomarkers for diagnosis. Exosomes are excellent drug delivery vehicles because they are endogenous carriers of molecular cargo, enhance targeting, improve biostability, and decrease cytotoxicity. Exosome-based medication delivery has been shown in preclinical studies to lessen systemic side effects and increase the effectiveness of traditional chemotherapy in the treatment of lung cancer. Furthermore, exosomes have unique applications for non-invasive liquid biopsies due to their strong resemblance to corresponding parent cells and their steady circulation in a variety of bodily fluids. Nevertheless, prior to the implementation of exosome-based delivery and diagnosis systems in clinical settings, several of related issues need to be addressed. Exosome isolation and detailed characterization are the first and most significant constraint. Clinical-grade exosome manufacturing and additional translation have been impeded by the absence of standardised exosome isolation protocols, storage strategies, and quality control measures. Furthermore, a significant barrier to clinical translation and cost containment in production is the bulk and scalable exosome manufacturing process. Both the abundance of exosomes and the availability of biofluids fluctuate often. To guarantee a steady and reliable supply of exosomes, a standardised procedure for exosome isolation is also necessary. Additionally, the exosome preparation needs to be of the highest caliber, homogenous, and uncontaminated by other cellular vesicles. The current obstacle in the creation of exosome biomarkers is the dearth of extensive prospective studies that can demonstrate the validity of exosome liquid biopsy as a substitute for tumour tissue biopsy. Maintaining appropriate manufacturing standards is the key issue with biologically-origin exosome therapy systems. Therefore, it will be necessary to establish standardised protocols to guarantee consistent exosome production prior to the clinical implementation of exosome-based therapy. Before exosomes be used in clinical applications, additional study must be done, even if exosome-mediated therapy, diagnosis, and prognosis seem promising.
